# Exploring the efficiency of nitrogenated carbon quantum dots/TiO_2_ S-scheme heterojunction in the photodegredation of ciprofloxacin in aqueous environments

**DOI:** 10.55730/1300-0527.3679

**Published:** 2024-03-11

**Authors:** Yılmaz ATEŞ, Zafer EROĞLU, Özkan AÇIŞLI, Önder METİN, Semra KARACA

**Affiliations:** 1 Department of Chemistry, Faculty of Science, Atatürk University, Erzurum, Turkiye; 2 Department of Chemistry, College of Sciences, Koç University, İstanbul, Turkiye; 3 Koç University Surface Science and Technology Center (KUYTAM), İstanbul, Turkiye

**Keywords:** Carbon quantum dots, green synthesis, TiO_2_, S-scheme heterojunction, photocatalyst, ciprofloxacin degradation

## Abstract

In this study, we developed a heterojunction photocatalyst, namely nitrogen-doped carbon quantum dots/titanium dioxide (*N-*CQDs/TiO_2_), for the effective and sustainable treatment of ciprofloxacin (CIP) antibiotic in aqueous solution. First, *N-*CQDs were prepared from a chitosan biopolymer with a green, facile, and effective hydrothermal carbonization technique and then anchored on the TiO_2_ surface via a hydrothermal process. The morphological, structural, and optical properties of the as-prepared materials were characterized by using advanced analytical techniques. The impacts of the mass percentage of *N*-CQDs, catalyst and CIP concentration, and pH on photocatalytic CIP degradation were investigated in depth. Comparative analyses were performed to evaluate different processes including adsorption, photolysis, and photocatalysis for the removal of CIP with TiO_2_ and *N-*CQDs/TiO_2_. The results revealed that *N*-CQDs/TiO_2_ exhibited the highest CIP removal efficiency of up to 83.91% within 120 min using UVA irradiation under optimized conditions (10 mg/L CIP, 0.4 g/L catalyst, and pH 5). Moreover, the carbon source used in the fabrication of *N*-CQDs was also considered, and lower removal efficiency was obtained when glucose was used as a carbon source instead of chitosan. This excellent improvement in CIP degradation was attributed to the ideal separation and migration of photogenerated carriers, strong redox capability, and high generation of reactive oxygen species provided by the successful construction of the *N-*CQDs/TiO_2_ S-scheme heterojunction. Scavenger experiments indicated that h^+^ and •OH reactive oxygen species were the predominant factors for CIP elimination in water. Overall, this study presents a green synthesis approach for *N*-CQDs/TiO_2_ heterojunction photocatalysts using natural materials, demonstrating potential as a cost-effective and efficient method for pharmaceutical degradation in water treatment applications.

## 1. Introduction

The intensive use of pharmaceutical compounds, such as various antibiotics and antiinflammatories, and the resulting increase in their release into the receiving environment has led to the emergence of a series of environmental problems, particularly in water [1–3]. Ciprofloxacin (CIP), a fluoroquinolone antibiotic derivative, has a wide range of utilization in the treatment of humans and animals, and even in low concentrations, residues of CIP antibiotic can provoke serious problems that threaten human health and aquatic ecosystems [3,4]. Because of the high bacterial resistance and lower biodegradability of sewage containing CIP, its removal from wastewater is of great importance for human health [3]. In this regard, to remove CIP and its metabolites from water, advanced oxidation processes (AOPs) such as sonocatalytic, photocatalytic, and Fenton processes have received much attention [3–5]. Semiconductor-based photocatalytic processes, recognized as environmentally friendly solutions, constitute a promising option for addressing water treatment challenges. It is anticipated that these methods, employing high-performance and eco-friendly catalysts, could serve as optimal solutions in the quest for efficient water treatment [6]. To achieve high photocatalytic performance in the presence of a semiconductor photocatalyst, variables such as sunlight-harvesting ability, high charge separation and transfer, and the occurrence of active sites for photoredox reactions are critical. Moreover, the preparation of such an effective photocatalyst from natural resources using simple and inexpensive methods is important for a sustainable environment [7]. Therefore, the synthesized catalyst has an important role in the success of photocatalytic processes. TiO_2_ has been the most preferred semiconductor due to its low environmental toxicity, excellent oxidation features, high chemical stability, and inexpensiveness [8]. Since the band gap of TiO_2_ is 3.2 to 3.5 eV, the production of photogenerated carriers responsible for its photocatalytic properties requires exposure to ultraviolet light [8,9]. Factors such as the low photocatalytic activity of TiO_2_ under sunlight and rapid charge recombination, causing a decrease in quantum efficiency, reduce the performance of TiO_2_ and its utilization as a photocatalyst alone does not produce satisfactory results [9,10].

To bypass these disadvantages and boost the photocatalytic performance of TiO_2_, strategies such as surface modification with metal and nonmetal elements, combinations with other semiconductors, and the design of multiple components are applied [11,12]. Recently, the combination of TiO_2_ with carbon quantum dots (CQDs) has been a favorite approach to enhance the light-harvesting ability and hence the photocatalytic activity [11]. CQDs are a new category of photoluminescent (PL) carbon nanomaterials with sizes of less than 10 nm, consisting of sp^2^/sp^3^ hybridized carbon atoms carrying different functional surface groups [12]. CQDs have gained increasing significance owing to valuable properties such as low toxicity, perfect electron transfer/reservoir characteristics, good upconverted photoluminescence behavior, chemical inertness, and superior biocompatibility [6,13,14]. Despite these unique properties of CQDs, however, their quantum efficiency is limited, and to increase this, doping with heteroatoms has recently attracted much attention [15]. In the literature, it is reported that when nitrogen is added to nanostructured carbon materials, charge delocalization improves, the carbon’s work function decreases, and photoluminescence emission capacity effectively increases. These approaches lead to CQDs with unique chemical and physical properties such as tunable electronic and optical properties [16]. Numerous investigations have employed CQDs/TiO_2_ as a photocatalyst for organic pollutant degradation [6–8,11–15]. However, there are still unresolved queries in this field, particularly regarding the impact of the carbon source material on the photocatalytic efficiency of TiO_2_.

Studies exploring the influence of different carbon sources on the photocatalytic performance of TiO_2_ are prevalent in the scientific literature. The utilization of renewable natural resources in the synthesis of CQDs attracts more attention than other synthetic materials because they are environmentally friendly. The synthesis of CQDs, which are used beneficially in many areas with simple and environmentally friendly methods without the use of synthetic chemicals, is attracting great attention in terms of green chemistry [17]. Chitosan is the *N*-deacetylated derivative of chitin, a renewable natural polysaccharide obtained from crab and shrimp. It is a suitable natural material for the synthesis of CQDs because it has abundant functional groups of −OH and −NH_2_, and it is biocompatible, natural, and nontoxic. It demonstrates diverse physical characteristics, including viscosity, adhesiveness, and potential solubility in a range of media [17–19]. In the literature, some studies have addressed the synthesis of CQDs from chitosan for diverse applications. Ni et al. [20] synthesized an 8-hydroxy-quinoline-7-carboxylic acid/TiO_2_ (HQC/TiO_2_) photocatalyst for phenol degradation under visible light illumination and utilized CQDs from chitosan to enhance the dynamic and cyclic stability of HQC/TiO_2_. In another study, Midya et al. [21] prepared a photocatalyst through in situ formation and accumulation of TiO_2_ nanoparticles and CQDs on the surface of cross-linked chitosan. They used this catalyst in the photooxidation of some organic compounds under solar light and obtained good photocatalytic performance. However, to the best of our knowledge, there is no research investigating the utilization of CQDs/TiO_2_ catalysts fabricated by combining CQDs derived from chitosan with TiO_2_ in photocatalytic applications.

In light of the above considerations, we fabricated an S-scheme *N-*CQDs/TiO_2_ heterojunction photocatalyst for the removal of CIP from water under UVA irradiation. The synthesis of *N-*CQDs/TiO_2_ was carried out by the hydrothermal method using chitosan as a precursor. Among many methods applied for the synthesis of CQDs, the hydrothermal method is a highly preferred strategy because it is convenient, low-cost, easy, and environmentally friendly [22]. Next, the impact of several operational parameters including catalyst concentration, CIP concentration, and initial solution pH on the photocatalytic efficacy of the *N-*CQDs/TiO_2_ was investigated. A potential photooxidation mechanism was proposed based on radical trapping experiments. The catalyst was synthesized through direct contact between TiO_2_ nanoparticles and chitosan-derived *N-*CQDs without the use of any mediator materials. This synthesis approach facilitated exceptional charge separation and transfer, resulting in significantly superior performance compared to pure TiO_2_.

## 2. Materials and methods

### 2.1. Fabrication of *N*-CQDs

The synthesis of chitosan-based *N-*CQDs was achieved using an efficient, simple, green, and one-step hydrothermal carbonization method, constituting a modified version of the method reported by Hazarika and Karak [23]. After adding 0.5 g of chitosan to 50 mL of 1 M acetic acid, the mixture was agitated for 10 min to produce a translucent sole. After adding 0.3 g of urea, the mixture was subjected to 30 min of ultrasonication (240 W/L output power; VWR Ultrasonic Cleaner USC-THD, VWR, Shanghai, China). The reaction mixture was then stirred for 10 min after adding 0.4 mL of glycerol and for an additional 1 h after adding 15 mL of 1 M HCl. The mixture was transferred to a 100-mL Teflon-lined stainless steel reactor and left to sit at 150 °C for 6 h. The reactor was cooled to room temperature following carbonization. After being removed from the reactor, the mixture was once again centrifuged (Universal 320 Hettich, Andreas Hettich GmbH, Tuttlingen, Germany) at 9000 rpm to separate the solid portion and passed through a 0.45-μm membrane filter. It was then stored in a sealed container at 5 °C for later use in experiments.

### 2.2. Fabrication of TiO_2_

A previously reported method developed by our group was used for TiO_2_ synthesis with minor modifications [7]. Experimental details regarding the synthesis of TiO_2_ are included in the Supporting Information.

### 2.3. Fabrication of *N*-CQDs/TiO_2_

The *N-*CQDs/TiO_2_ nanophotocatalyst was prepared using a five-step protocol as follows: Step 1- Adding 40 mL of water to 10 mL of the *N*-CQDs solution prepared as described above and stirring for 15 min in a magnetic stirrer. Step 2- Dropwise addition of titanium(IV) ethoxide of 1.6 mL to the solution prepared in the first step and mixing in a magnetic stirrer for 1 h. Step 3- Carbonization of the mixture taken into the Teflon-lined stainless steel reactor in a muffle oven (Lenton, Hope Valley, UK) at 150 °C for 6 h. Step 4- Separating the suspended *N-*CQDs/TiO_2_ nanoparticles taken out of the reactor and washing by centrifuging with ethanol for 10 min at 9000 rpm. Step 5- Drying the nanoparticles obtained in Step 4 by heating them at 50 °C for 8 h and storing them in a closed container for subsequent use. The pathway followed while synthesizing the catalyst is schematized in Figure S1. The characteristics of CIP chosen as the model pollutant are listed in Table S1.

## 3. Results and discussion

### 3.1. Catalyst characterization

The *N*-CQDs/TiO_2_ heterojunction photocatalyst was fabricated using hydrothermal treatment of a chitosan biopolymer as a natural carbon source in a mixture of glycerol, urea, water, and concentrated HCl at 150 °C for 6h, as schematized in Figure S1. Bare TiO_2_ nanoparticles were also prepared by the same strategy without the addition of *N*-CQDs. As-prepared samples were characterized by X-ray diffraction (XRD), transmission electron microscopy (TEM), scanning electron microscope/energy-dispersive X-ray spectroscopy (SEM/EDS), Fourier transform infrared spectroscopy (FTIR), and X-ray photoelectron spectroscopy (XPS) analyses.

**Figure 1:**
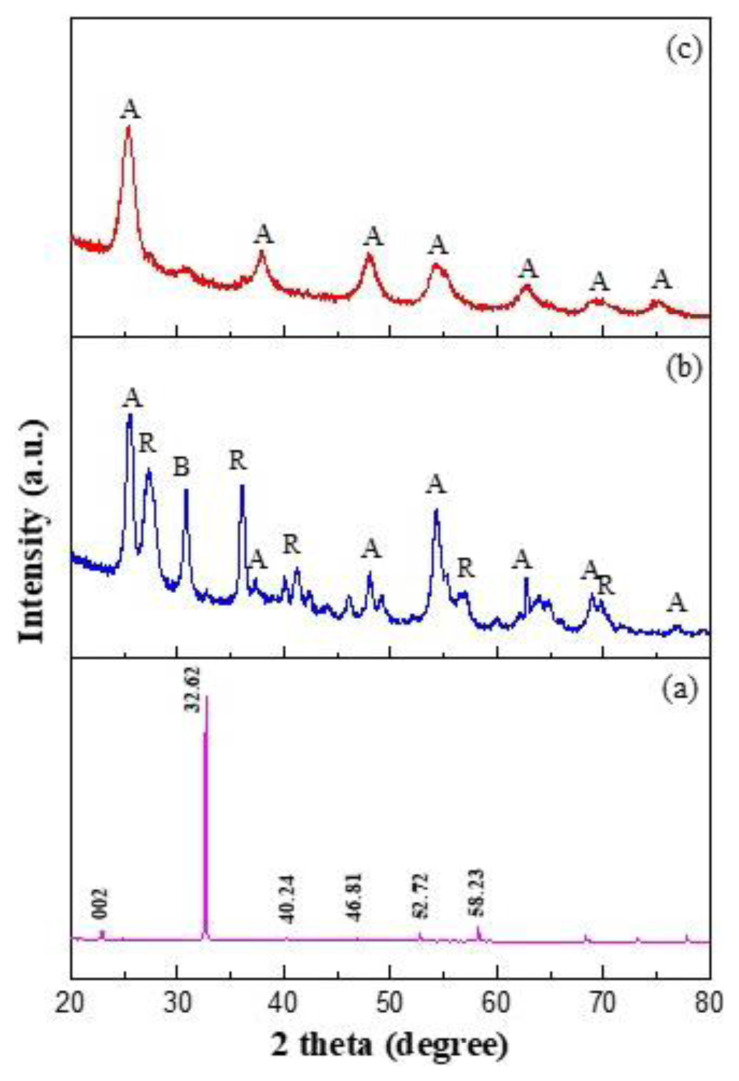
XRD patterns of a) *N-*CQDs, b) TiO_2_, and c) *N-*CQDs/TiO_2_ nanocomposite.

The preparation procedure and compositional variation of the prepared samples were monitored by powder XRD analysis. As illustrated in Figure 1a, a sharp peak position centered at 22.91° of the *N*-CQDs was ascribed to the (002) lattice plane of graphite, and the determined interlayer spacing of 0.39 nm was wider than the graphitic interlayer distance (0.32 nm) [24]. The enlargement in interlayer distance was caused by the formation of more oxygenated functional groups such as -COOH, -OH, and amine groups on the surface and edges of *N*-CQDs during the hydrothermal process [25]. Additionally, the sharp peak at 2θ = 32.62° was attributed to irregular graphite-like *N-*CQDs [26]. The noticeable peak at 2θ = 40.24° (100) could be indexed as graphitic sp^2^ carbon clusters, while the other peaks at 58.23° (103) and 68.37° (220) signified a diamond-like sp^3^ hybridized carbon structure [27]. Moreover, the peaks at 2θ = 46.81° (101) and 52.72° (102) were indexed to the diffraction pattern of graphitic carbon, representing conjugated sp^2^ carbon scaffolds [24,27,28]. These results are in line with those previously published for CQDs [27,29,30]. From the XRD diffractogram of TiO_2_ (Figure 1b), it was determined that the sample included both rutile and anatase phases, parallel to the outcomes reported by Wang et al [31]. The distinctive diffraction peaks of TiO_2_ at 25.32° (101), 37.39° (004), 48.03° (200), 54.32° (105), 62.75° (204), 68.88° (116), and 77.01° (215) overlapped well with the characteristic diffraction pattern of the anatase phase in the tetragonal crystal structure (JCPDS No. 21-1272) [32,33]. In Figure 1b, the 2θ diffraction peaks located at 27.53° (110), 36.05° (101), 41.31° (111), 57.12° (220), and 69.79° (301) confirm the presence of the rutile phase of TiO_2_ (JCPDS Card 00-21-1276) [34,35]. Additionally, the peak observed at 2θ = 30.80° indicates the brookite phase of TiO_2_ (JCPDS No. 84-1750) [7]. From the XRD graph of the *N*-CQDs/TiO_2_ nanocomposites (Figure 1c), only peaks belonging to the anatase phase of TiO_2_ were observed. The absence of a rutile phase in the *N*-CQDs/TiO_2_ nanocomposites can be attributed to the fact that the carbon content prevents a crystal transformation of the crystal phase of TiO_2_ to form the rutile phase [6]. The XRD data revealed that the *N*-CQDs were successfully assembled onto the TiO_2_ surface to yield *N*-CQDs/TiO_2_ composites. The disappearance of the *N-*CQDs peaks in the XRD diffractogram of the *N*-CQDs/TiO_2_ nanocomposites can be clarified by the weak crystallinity, low quantity, and uniform distribution of *N*-CQDs in the nanocomposite structure [6]. The average crystal sizes were calculated to be 14.88 nm and 8.09 nm for bare TiO_2_ and *N*-CQDs/TiO_2_, respectively, using the Scherrer equation based on the anatase (101) diffraction peak at 2θ = 25.32° with lattice spacing of 0.36 nm [36].

**Figure 2:**
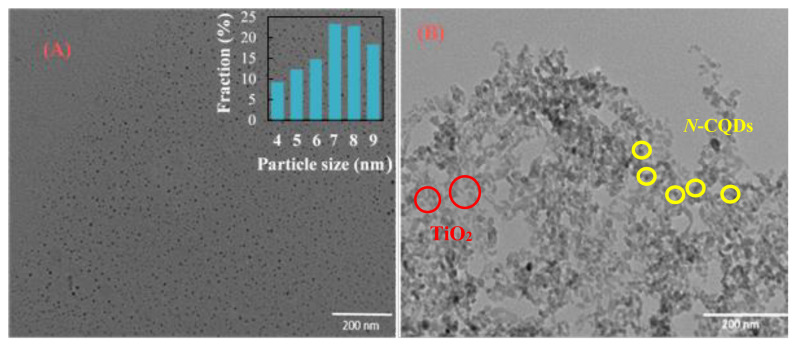
Representative TEM images of (A) *N-*CQDs and (B) *N-*CQDs/TiO_2_ heterojunction photocatalyst

Morphological analyses of the *N-*CQDs and *N*-CQDs/TiO_2_ nanocomposites were performed by TEM. The presence of spherical nanoparticles with average size of about 7–8 nm in the TEM image in Figure 2a verifies that *N*-CQDs were successfully synthesized from the chitosan by the hydrothermal method. Figure 2b shows that the *N*-CQDs were uniformly disseminated on the surfaces of TiO_2_ particles with dimensions of about 8 nm.

SEM analysis was conducted to examine the surface morphology of the *N-*CQDs, bare TiO_2_ nanoparticles, and *N-*CQDs/TiO_2_ nanocomposites (Figure S2). From the SEM image of *N*-CQDs, it is noteworthy that the *N-*CQDs are partially singular and mostly form separate phases as aggregates (Figures S2A and S2B). In Figure S2C, it is seen that there are TiO_2_ nanoparticles with aggregated spherical-like shape. Additionally, it was clearly observed that the typical TiO_2_ morphology did not change after the introduction of *N*-CQDs into the structure, but it shrank in size (Figure S2D). This allowed the catalyst surface to increase and offered a more reactive area, which is beneficial in photocatalytic degradation. Elemental compositions of as-prepared samples were determined from EDX data (Figure S2E). By using EDX tests, it was determined that the *N*-CQDs sample had 35.39 wt.% C, 20.31 wt.% O, and 12.57 wt.% N; the TiO_2_ sample had 51.02 wt.% Ti and 48.98 wt.% O; and the *N*-CQDs/TiO_2_ nanocomposite sample had 4.87 wt.% C, 49.14 wt.% O, 43.63 wt.% Ti, and 0.06 wt.% N. These results clearly confirm the distribution of *N-*CQDs on the TiO_2_ surface and the successful fabrication of *N*-CQDs/TiO_2_ nanocomposites.

**Figure 3:**
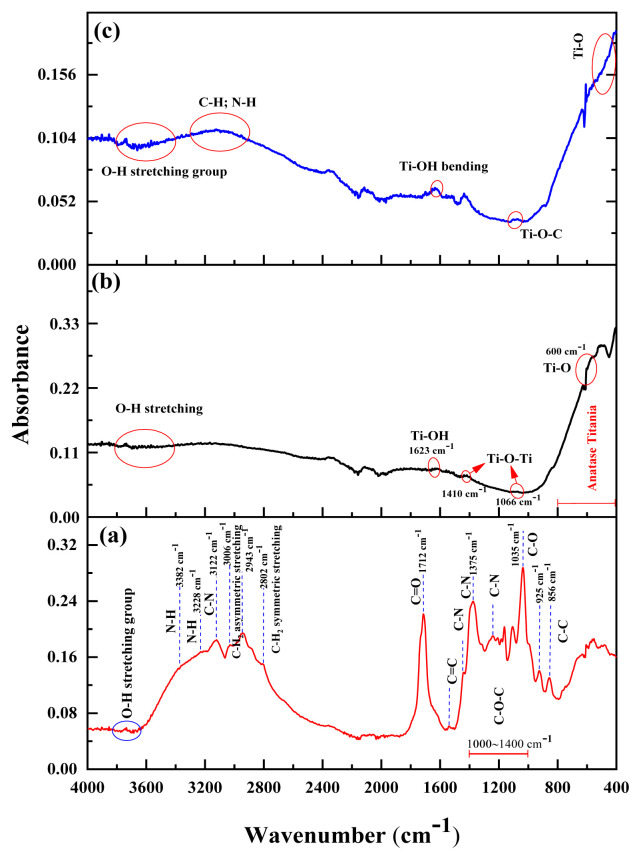
FTIR spectra of (a) as-prepared *N-*CQDs, (b) TiO_2_, and (c) *N-*CQDs/TiO_2_ nanocomposite, respectively.

The absorption bands and related functional groups in the *N*-CQDs, TiO_2_, and *N*-CQDs/TiO_2_ samples were investigated using FTIR analysis. The resulting spectra are presented in Figure 3. In Figure 3a, the FTIR spectrum of the *N*-CQDs shows an important peak at 1712 cm^−1^ and a broad peak between 3200 and 3600 cm^−1^, which correspond to C=O and amino groups/O–H stretching vibrations, respectively [37,38]. It can be inferred that the peaks at 1375, 1035, 2802, and 3006 cm^−1^ are responsible for the C–N, C–O, C-H_2_ symmetric stretching, and C-H_2_ asymmetric stretching vibrations, respectively, while the peak at 3382 may be associated with the N–H vibrations [38,39]. A graphitic assembly and an unsaturated aromatic ring may have formed during the hydrothermal treatment process according to the stretching vibration peak of C=C at 1544 cm^−1^ [38]. The distinct peaks at 1255 cm^−1^, 1442 cm^−1^, and 1375 cm^−1^ correspond to the stretching vibration modes of C-N heterocycles, whereas the peak at 3228 cm^−1^ represents the NH stretching vibrations [38]. The absorption band at 2943 cm^−1^ was assumed to be the asymmetric stretching vibration of −CH_2_ [39,40]. According to FTIR results, hydrophilic functional groups like -COOH, -NH_2_, and -OH coated the surface of the *N*-CQDs. Additionally, the results implied that the exceptional solubility of *N*-CQDs in solution played a role. The FTIR spectra of TiO_2_ and *N*-CQDs/TiO_2_ are shown in Figures 3b and 3c, respectively. Both samples exhibited a broad absorption band below 1000 cm^−1^, indicative of the Ti-O-Ti bond’s vibration. The O-H stretching vibration of the adsorbed water on the sample’s surfaces was responsible for the broad absorption band observed at approximately 3200 cm^−1^ and Ti-OH bending vibrations were observed at 1623 cm^−1^ for the two samples [41]. The bonds of C-O-C, Ti-O-C, and Ti-O-Ti were responsible for the intense peaks of the *N*-CQDs at 1000–1400 cm^−1^, the peak of the *N*-CQDs/TiO_2_ at 1060 cm^−1^, and the bands at 1066 and 1410 cm^−1^ for TiO_2_, respectively [41,42]. These findings support the XPS results. Furthermore, compared to bare TiO_2_, it was found that the broad absorption band below 1000 cm^−1^ widened and shifted toward a high wavenumber in the FTIR spectra of the *N*-CQDs/TiO_2_ nanocomposites. This behavior was linked to a combination of Ti-O-Ti and Ti-O-C vibrations, indicating that the Ti-O-C bond formation was responsible for the coupling between bare TiO_2_ and *N-*CQDs [41,43]. The movement of the absorption band appeared from 611.39 cm^−1^ in the FTIR spectra of TiO_2_, resulting from the Ti-O vibration, to 611.37 cm^−1^ in *N*-CQDs/TiO_2_, clearly confirming that carbonaceous groups were incorporated on the surface of TiO_2_ [7].

**Figure 4:**
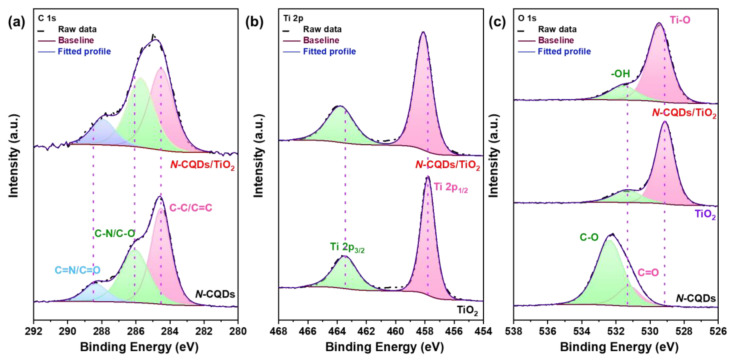
(a) High-resolution C 1s XPS spectra of *N-*CQDs and *N-*CQDs/TiO_2_ nanocomposites; (b) high-resolution Ti 2p XPS spectra of TiO_2_ and *N-*CQDs/TiO_2_ nanocomposites; (c) high-resolution O 1s XPS spectra of *N-*CQDs, TiO_2_, and *N-*CQDs/TiO_2_ nanocomposites.

The surface chemical composition of the prepared *N-*CQDs and *N-*CQDs/TiO_2_ nanocomposites and the interactions between *N-*CQDs and TiO_2_ were analyzed by XPS as displayed in Figures S3a and S3b and Figures 4a–4c. According to the XPS survey spectrum shown in Figure S3a, the *N-*CQD sample involved C, O, and N elements with binding energy peaks located at 285.08, 532.08, and 401.05 eV, respectively, revealing the successful synthesis of *N*-CQDs by the hydrothermal method. In the XPS survey spectrum of *N*-CQDs/TiO_2_ (Figure S3a), there were peaks at 285.07, 398.08, 458.08, and 530.09 eV belonging to C 1s, N 1s, Ti 2p, and O 1s, indicating the introduction of *N*-CQDs into the TiO_2_ structure. The high-resolution XPS spectrum of the N 1s region shows a peak at 401.05 eV (Figure S3b) that can be assigned to pyridine groups that have powerful electron-donating potential and provide excellent catalytic performance in redox reactions [14]. In the C 1s deconvoluted spectra of the *N*-CQDs (Figure 4a), the peaks at 284.5, 286.1, and 288.4 eV are assigned to C-C/C=C, C-N/C-O, and C=N/C=O bonds, respectively [44,45]. In the high-resolution C 1s spectrum of the *N*-CQDs/TiO_2_ nanocomposites given in Figure 4a, it was seen that the binding energy of the C 1s peaks changed to 284.5, 285.7, and 287.9 eV, respectively. This change in binding energies of C 1s peaks may indicate that the interaction between TiO_2_ and *N*-CQDs occurred through the Ti-O-C bonds formed between the C=O bonds in *N*-CQDs and Ti-O bonds in TiO_2_ [14,45]. For the pristine TiO_2_ (Figure 4b), the deconvolution of the Ti 2p signal fit into two peaks at 457.8 and 463.7 eV, assigned to the Ti 2p_3/2_ and Ti 2p_1/2_ core levels of the Ti^4+^ species, respectively, depicting a characteristic spin-orbital doublet splitting of 5.7 eV [45,46]. It was observed that these binding energies shifted to 458.2 and 463.9 eV in the *N*-CQDs/TiO_2_ nanocomposites, suggesting that TiO_2_ and *N*-CQDs may interact through the formation of Ti-O-C bonds [14]. In addition, in the high-resolution O 1s spectrum of the *N*-CQDs (Figure 4c), the two peaks located at 531.2 eV and 532.4 eV indicated the presence of C=O and C-O bonds [45]. The O1s spectrum of pristine TiO_2_ in Figure 4 c presents two pronounced peaks positioned at 529.1 eV and 531.4eV, which could be attributed to Ti-O, and C-O-H, respectively [46]. For the *N-*CQDs/TiO_2_, the binding energies associated with these bonds shifted to the area of higher energy at 529.45 eV and 531.59 eV, respectively. Compared with pristine TiO_2_, the binding energy of the Ti-O bond in the *N-*CQDs/TiO_2_ nanocomposites shifted towards the area of higher energy, showing that there was a charge transfer between the TiO_2_ and *N-*CQDs (Figure 4c) [46].

The textural properties and porosity of the prepared *N-*CQDs, TiO_2_, and *N-*CQDs/TiO_2_ nanocomposites were examined by the Brunauer–Emmett–Teller (BET) method. Figure S4A displays the nitrogen adsorption*-*desorption isotherms of the *N-*CQDs, TiO_2_, and *N-*CQDs/TiO_2_ nanocomposites. Their related Barrett–Joyner–Halenda (BJH) pore size distribution curves are illustrated in Figure S4B, and Table S2 summarizes the detailed textural properties of the catalysts. Concerning the IUPAC classification, all of the adsorption isotherms are of type IV isotherm exhibiting mesoporous character [47]. Incidentally, *N*-CQDs/TiO_2_ showed an H2-type hysteresis loop in p/p^0^ of 0.4–0.80, which corresponds to a wide pore size distribution or pores with narrow necks and wide bodies, referred to as “ink bottle pores” [47–50]. The isotherms belonging to TiO_2_ and the *N-*CQDs presented a type H3 hysteresis loop, which does not exhibit limiting adsorptions at high p/p^0^ values, implying the existence of slit-shaped pores [50]. The shifting of the inflection point to lower pressures for *N-*CQDs/TiO_2_ nanocomposites compared to that of TiO_2_ signified a decrease in pore size as a result of *N-*CQDs incorporated into the TiO_2_ structure [49], which was evidenced by the pore size distribution of the same samples as represented in the BJH plot and Table S2. This implies that there are strong interactions between *N*-CQDs and TiO_2_ nanoparticles. As can be seen from Table S2, the respective BET surface areas of the TiO_2_, *N-*CQDs, and *N-*CQDs/TiO_2_ photocatalysts were estimated to be 71.798, 1.091, and 213.792 m^2^/g. This could be attributed to the shrinkage of the crystal size of TiO_2_, as supported by XRD and TEM results, and the formation of narrow pores as a result of the arrangement in the pore structure with the introduction of *N-*CQDs into the TiO_2_ structure. Pore volumes of catalysts in the same order were found to be 0.186, 0.003, and 0.203 cm^3^/g. On the contrary, the mean pore diameter of the *N-*CQDs/TiO_2_ (3.210 nm) was much narrower than that of the TiO_2_ (9.524 nm) and *N*-CQDs (5.171 nm). The increase in pore volume and surface area of *N-*CQDs/TiO_2_ compared to TiO_2_ means more active centers, which helps to raise the CIP concentration of the *N-*CQDs/TiO_2_ surface, simplifying the reaction between reactive oxygen species (ROS) and CIP molecules [51,52]. In photocatalytic processes, adsorption occurs before degradation, which requires a high surface area [52]. However, it cannot be said that there is a direct relationship between the improved photocatalytic efficiency and the surface area [52]. The adsorption of the pollutant, together with its degradation products and ROS, to the catalyst’s surface is the initial stage in heterogeneous photocatalytic reactions. Therefore, the catalyst’s surface area plays a crucial role in supplying active centers that are appropriate for adsorption. However, since there will not be any accumulation on the catalyst surface, there is no direct correlation between the size of the catalyst surface and removal effectiveness, because the rate at which the ROS degrade pollutant molecules is higher than the rate at which they adsorb them. It is crucial that ROS arise without charge-carrier recombination and that redox reactions take place between these species and pollution molecules.

**Figure 5:**
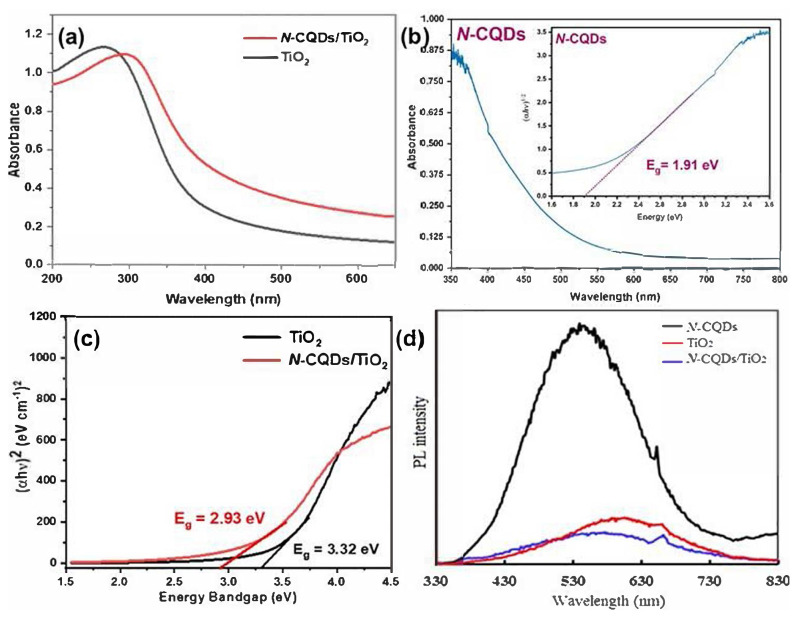
UV-Vis-DRS spectra of (a) TiO_2_ and *N-*CQDs/TiO_2_ nanocomposites and (b) *N*-CQDs, with the inset of (b) showing the band gap energy (E_g_) of the as-prepared *N*-CQDs; (c) Tauc plots of TiO_2_ and *N-*CQDs/TiO_2_ nanocomposites; (d) PL emission spectra at excitation wavelength of 325 nm for as-prepared *N-*CQDs, TiO_2_, and *N-*CQDs/TiO_2_ nanocomposites.

The effectiveness of a photocatalyst significantly depends on its ability to harvest light and prevent charge recombination, as well as its efficiency in charge separation. Therefore, UV-Vis-NIR DRS measurements of *N-*CQDs, TiO_2_, and *N-*CQDs/TiO_2_ nanocomposites were performed; band gaps were calculated and photoluminescence spectra were obtained to evaluate the improvement in the photocatalytic activity of TiO_2_ with the introduction of *N-*CQDs into the TiO_2_ structure. The results are collectively presented in Figure 5. As demonstrated in Figure 5a, TiO_2_ absorbs only in the UV region, while *N-*CQDs/TiO_2_ absorbs in both the UV and visible regions due to the presence of *N-*CQDs, whose absorption band is red-shifted. The shifting of the absorption edge of *N-*CQDs/TiO_2_ (423 nm) to the more visible region compared to that of TiO_2_ (373 nm) can be attributed to the chemical interactions of TiO_2_ and *N-*CQDs through the Ti-O-C bonds. Possible interactions in the *N-*CQDs/TiO_2_ nanocomposites affect the interfacial transport rate of e^−^/h^+^ pairs, which is highly beneficial for catalytic activity [51,53]. Figure 5b shows the absorption spectrum and band gap energy of *N*-CQDs (see inset). The typical peak at 350 nm resulting from the n → π* transition of the C=O bond and other functional groups reveals that the synthesis of *N-*CQDs was successfully achieved, similar to other published reports on *N*-CQDs [54–56].

The band gap energy (E_g_) of the *N-*CQDs, TiO_2_, and *N-*CQDs/TiO_2_ nanocomposites was estimated using the Tauc formula (Eq. 1) [51,52]:


(1) $${\left(\alpha h\nu \right)}^{2}=A(h\nu -E_{g})$$

Here, h, ν, α, E_g_, and Asymbolize the Planck constant, frequency of vibration, absorption coefficient, band gap, and a proportional constant, respectively. The estimated band gap values of the samples are given in Figures 5b and 5c. The E_g_ values of the *N*-CQDs, pristine TiO_2_, and *N-*CQDs/TiO_2_ nanocomposites were computed to be 1.91, 3.32, and 2.93 eV, respectively. The reduction of the band gap from 3.32 eV to 2.93 eV reveals that the *N-*CQDs/TiO_2_ nanocomposites could benefit from all wavelengths. Accordingly, their photocatalytic activity will be higher than that of TiO_2_ [51].

For a better understanding of the role of *N-*CQDs in the capability of effectual charge transport and separation in the *N-*CQDs/TiO_2_ photocatalyst, the PL spectra of the *N-*CQDs, TiO_2_, and *N-*CQDs/TiO_2_ nanocomposites were recorded at an excitation wavelength of 325 nm at room temperature. The *N-*CQDs exhibited the most powerful PL emission spectrum, centered at 539.5 nm. After coupling with TiO_2_ nanoparticles, the *N-*CQDs/TiO_2_ nanocomposites displayed the weakest PL intensity (Figure 5d), attributing to the limited recombination of photogenerated e^−^/h^+^ pairs, probably due to the formation of the binary heterojunction between *N*-CQDs and TiO_2_ [51,57].

### 3.2. Comparison of different processes for CIP removal

**Figure 6:**
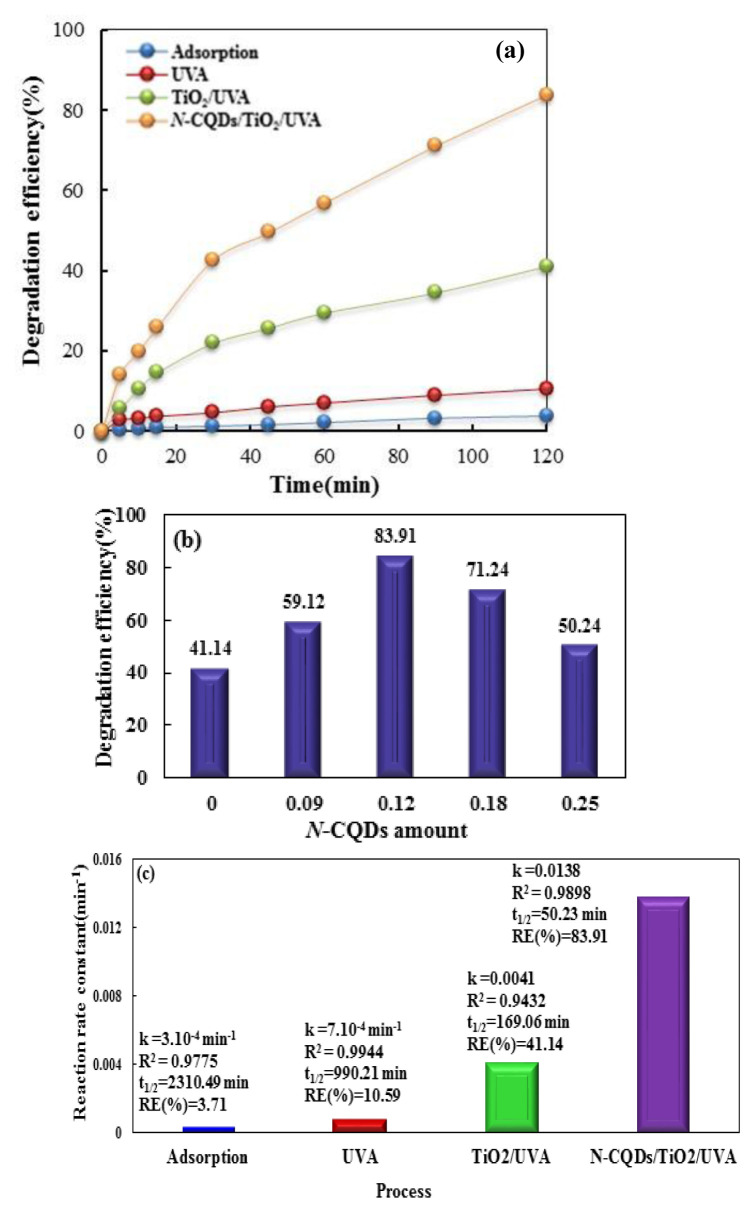
(a) Effects of different processes on the removal efficiency of CIP; (b) impact of *N-*CQD amount on CIP degradation using the *N*-CQDs/TiO_2_ nanophotocatalyst; (c) degradation efficiencies and kinetic parameters for CIP degradation via different processes. Experimental conditions: [Catalyst]_0_ = 0.4 g/L, [CIP]_0_ = 10 mg /L, and pH = 5.

To evaluate the contribution of each considered process to CIP elimination in the *N-*CQDs/TiO_2_/aqueous CIP solution system, several experiments were performed under predetermined optimum conditions of 10 mg/L CIP, 0.4 g/L catalyst, and pH 5 (natural pH). Figure 6a illustrates the results of a comparative study on CIP removal. As can be seen from Figure 6a, the single application of adsorption and photolysis (UVA) processes for CIP removal resulted in CIP removal of 3.38% and 10.58% after 120 min of irradiation time, respectively. In other words, these treatment methods were insufficient in removing CIP due to the lack of adsorption capacity of the *N-*CQDs/TiO_2_ photocatalyst or unsatisfactory free radical production via UVA irradiation. On the other hand, the performance of TiO_2_/UV was significantly higher than that of UVA irradiation alone, attaining 41.14% CIP removal and revealing the efficient role of TiO_2_ as a catalyst that contributes to the production of free radicals through the photocatalytic process. When TiO_2_ was combined with *N-*CQDs, 83.91% CIP removal was achieved. The higher degradation of CIP while using the *N-*CQDs/TiO_2_ photocatalyst compared to the TiO_2_ catalyst showed that the combination of TiO_2_ with *N-*CQDs can improve the photocatalytic activity under UVA irradiation. This enhancement in the presence of *N*-CQDs, attributed to the charge transfer occurring at the interface between *N-*CQDs and TiO_2_, led to the improved photocatalytic efficiency of the *N*-CQDs/TiO_2_ nanocomposites and enhanced the CIP degradation [8].

The carbon content of the composite is important in the photocatalytic performance of semiconductors equipped with *N*-CQDs. The appropriate amount of carbon for the *N-*CQDs/TiO_2_ catalyst was found by keeping the TiO_2_ ratio constant and changing the *N*-CQDs amounts. Figure 6b shows the results obtained from the experiments. As seen from Figure 6b, the removal efficiencies for 0, 0.09, 0.12, 0.18, and 0.25 g of *N*-CQDs were found to be 41.14%, 59.12%, 83.91%, 71.27%, and 50.24%, respectively. It can be understood from the results that the best charge transfer was obtained by using 0.12 g of *N-*CQDs. When there was an appropriate proportion of *N-*CQDs in the composite, the *N-*CQDs uniformly distributed on the TiO_2_ surface acted as both acceptors and donors to create a new electric field. In this way, the charge carriers were separated, the recombination tendency was reduced, and redox reactions of nanocomposites were stimulated by e^−^/h^+^ pairs. Therefore, it caused an increase in ROS, which increased CIP removal. On the other hand, *N*-CQDs increased the light absorption of TiO_2_ nanoparticles due to their spectral properties, which increased CIP removal efficiency. Moreover, the *N-*CQDs provided active centers suitable for adsorption, resulting in an increase in the amount of adsorbed species [41,57]. Increasing the amount of *N-*CQDs above 0.12 g caused a decrease in CIP removal efficiency. In this case, the excess *N-*CQDs competed with TiO_2_ to absorb the incident light. Moreover, upon burying a large part of the TiO_2_ surface under *N-*CQDs, the photoexcitation of TiO_2_ decreased and the photocatalytic efficiency of the catalyst also decreased because the amount of charge carriers decreased. Additionally, an excess of *N*-CQDs caused light scattering. On the other hand, the abundance of *N-*CQDs provided recombination centers for light-induced e^−^/h^+^ pairs. All of these outcomes resulted in decreased photocatalytic activity [41,58]. The experimental data obtained for each process were applied to the pseudo-first-order model using the following equations [7]:


(2) $$ln\frac{A_{0}}{A}=k_{app}t$$

(3) $$t_{1/2}=\frac{\text{ln}\;2}{k_{app}}$$

Here, A_0_ and A_t_ denote the CIP absorbance value before photocatalytic oxidation and after some certain time (min), respectively; k_app_ is the rate constant (apparent); and t is the time [57,58]. The fitted first-order equation-related kinetic parameters of the experimental data, namely k (min^−1^) and R^2^, together with the calculated t_1/2_ (min) value, are shown in Figure 6c. The analysis results showed that CIP removal conformed to the pseudo-first-order kinetic model for all processes. The photocatalytic process using *N*-CQDs/TiO_2_ nanocomposites with the highest k_app_ (0.0138 min^−1^) and lowest t_1/2_ (50.23 min) was considered the best-performing process [7].

It is obvious that doping with *N-*CQDs plays a major role in the performance of *N-*CQDs/TiO_2_ in CIP removal. However, in order to see the effect of the carbon source on the performance of *N*-CQDs, we prepared *N-*CQDs from glucose using the same conditions described for chitosan. When CIP removal was examined under the same conditions, 39% removal efficiency in 120 min was obtained with the catalyst prepared from glucose. The fact that the *N*-CQDs/TiO_2_ nanocomposites prepared with *N-*CQDs obtained from chitosan showed much better performance than the catalyst prepared from glucose may be due to the different functional groups and chain lengths that the two sources possess [59]. This can be explained by the fact that *N*-CQDs generated from chitosan, as opposed to those derived from glucose, have richer surface functional groups due to the presence of N groups, which enhance CIP adsorption and encourage photocatalytic activity. Based upon this result, it was concluded that chitosan is a suitable precursor for the synthesis of *N*-CQDs.

### 3.3. Effect of operational parameters on the photocatalytic degradation of ciprofloxacin in the presence of *N*-CQDs/TiO_2_ nanocomposites

#### 3.3.1. Catalyst amount

To find the optimum catalyst dosage value in the photocatalytic oxidation of CIP, experiments were conducted at varying catalyst concentrations in the range of 0.05–0.6 g/L while other operational parameters were constant (CIP concentration of 10 mg/L and pH 5). As revealed in Figure S5, CIP degradation efficiency increased from 42.58% to 83.91% in 120 min upon increasing the catalyst concentration from 0.05 g/L to 0.40 g/L and then decreased thereafter. The enhancement in degradation efficiency can be explained by the larger number of reachable reaction centers on the *N-*CQDs/TiO_2_ surface and more generated free radicals being available for CIP degradation. It can be said that above a catalyst concentration of 0.4 g/L, a possible catalyst agglomeration resulting in a reduction of the active sites of the catalyst in solution would lead to a decrease in photocatalytic activity [60]. In addition, the increase in the turbidity of the solution with higher dosages of the catalyst leads to a decrease in the penetration of light through the solution, resulting in lower photocatalytic degradation [61]. Based on these outcomes, a concentration of 0.4 g/L was selected for the *N-*CQDs/TiO_2_ in all remaining steps of this study.

#### 3.3.2. Influence of initial concentration of CIP

In photocatalytic processes, the concentration of wastewater contaminated with antibiotics is an important parameter in pollution removal. To examine the effect of the concentration of CIP, it was considered in the range of 5–25 mg/L in this study. At all concentrations, the degradation efficiency of CIP was found to increase with increasing time (Figure S6). According to Figure S6, for residence time of 120 min, the degradation efficiency increased from 38.44% to 91.8%, reducing the initial CIP concentration from 25 to 5 mg/L. The decrease in removal efficiency as CIP concentration increased can be attributed to multiple factors. First, the fixed amount of ROS produced by a unit amount of catalyst was not enough to degrade the increasing concentration of CIP. Second, a concentrated pollutant solution could prevent *N*-CQDs/TiO_2_ particles from absorbing erased UVA waves [57,62].

#### 3.3.3. Impact of pH

Initial solution pH is an important parameter that influences the pollutant ionization degree, surface charge of the catalyst, radical production, and interfacial potential in AOPs [63]. Therefore, the effect of variation in the solution pH on CIP degradation efficiency in the presence of the *N-*CQDs/TiO_2_ photocatalyst was studied and zeta potentials of solid particles in catalyst/water suspensions were measured at different initial pH values (2, 3, 4, 5, 6, 8, 9, and 10). The results are given in Figures S7a and S7b. As clearly seen from Figure S7a, CIP degradation efficiencies of 19.28%, 41.75%, 55.02%, 83.91%, 61.93%, 64.28%, 65.71%, and 60.92% were obtained for the respective pH values of 2, 3, 4, 5, 6, 8, 9, and 10 for elapsed time of 120 min. The value of pH_zpc_ (i.e., zero point of charge) for the *N-*CQDs/TiO_2_ photocatalyst was determined to be 6.6 (Figure S7b). Below and above the pH_zpc_, the surface of the catalyst is positively and negatively charged, respectively. CIP has two pKa values (5.9 and 8.89), and it is present in aqueous solutions as a cation (CIP^0,+)^ below pH 5.9, as a zwitterion (CIP^−,+^) between pH 5.9 and pH 8.89, and as an anion (CIP^−,0^) above pH 8.89 [61,64]. As can be understood from Figure S7a, the highest percentage degradation of CIP was obtained at pH 5 (i.e., the natural pH of CIP), and then it decreased. The low degradation efficiency observed at values below pH 5 can be ascribed to the repulsive forces between the CIP molecules and the catalyst particles, both of which are positively charged, and the scavenger effect of Cl^−^ ions from the HCl used to adjust the pH of the solution. Although both the catalyst and the CIP molecules are positively charged at pH 5, the high degradation efficiency observed can be attributed to CIP adsorption by hydrogen bonds between the carboxyl, ketone, amine, and F groups of the CIP molecules and the catalyst molecules. It can be said that the presence of repulsive forces between catalyst particles whose surfaces become negative and CIP molecules causes a gradual decrease in CIP degradation after pH 5, resulting in low photocatalytic activity. Considering the points explained above, all experiments in this study were carried out at pH 5, which was the natural pH value of the CIP solution.

### 3.4. Band alignments

Prior to proposing a plausible mechanism for any photodegradation mechanism, it is necessary to define the band edges of the components within the constructed heterojunction. To satisfy this requirement, analytical methods such as valence band (VB)-XPS analyses and Tauc plots were used for the precise determination of photophysical features. The VB-XPS-measured VB potentials, as illustrated in Figures S8a and S8b, were identified where the tangent and oblique lines near the X-axis intersected at a point. The energy values of 2.55 eV for *N*-CQDs and 1.70 eV for TiO_2_ were extracted from the intersection of these critical findings and then standardized to the standard hydrogen electrode potential (E_VB-NHE_) using a well-known formula [65]:


(4) $$E_{VB-NHE}=\Phi +E_{VB-XPS}-4.44$$

Here, E_NHE_, Φ, and E_VB-XPS_ stand for the standard electrode potential, the XPS analyzer’s work function that is used herein, and the VB value obtained from VB-XPS analysis, respectively [66]. Utilizing this formula, VB values of 2.55 V for *N*-CQDs and 1.70 V for TiO_2_ were found. Upon combining the VB-XPS and Tauc plot outcomes, it was evident that the conduction bands (CBs) of the *N*-CQDs and TiO_2_ were positioned at 0.64 V and −1.62 V, respectively, as graphically depicted in Figure S8c.

**Figure 7:**
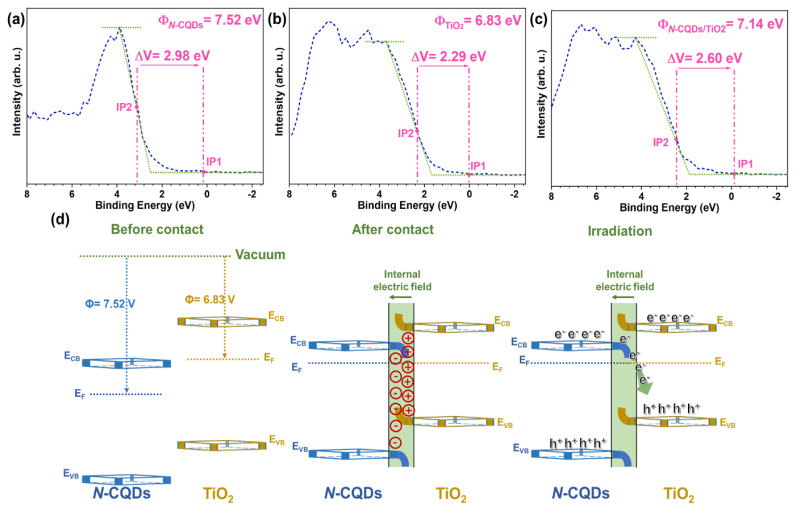
Work functions of (a) *N*-CQDs, (b) TiO_2_, (c) *N*-CQDs/TiO_2_, and (d) IEF between semiconductors before and after contact and under irradiation, respectively, and the resultant bending of band edges for *N*-CQDs and TiO_2_.

Investigating the photodegradation mechanism of CIP calls for an in-depth exploration of charge transfer dynamics at the interfaces between its components, a parameter of profound importance alongside band alignments. The functional roles of these band edges become clearer through the work functions of pristine materials obtained from both experimental and theoretical analyses. Using VB-XPS, we determined work function values for *N*-CQDs, TiO_2_, and the *N*-CQDs/TiO_2_ composite, as illustrated in Figures 7a–7c. When materials meet at their interfaces, the degree of the work function largely dictates the direction of the generated charge transfer. A greater work function value signifies that the Fermi level is more distant from the vacuum level, facilitating the acceptance of electrons by a material with a lower work function. Consequently, during this charge transfer, one component’s surface becomes positively charged while that of the other becomes negatively charged [67]. We determined the work function of each element by utilizing the following equation:


(5) ΔV=Φ-ϕ

Here, Φ is the work function of the material and ϕ is the work function of the device that is used (4.543 eV) [68]. The binding energy gap, ΔV, can be calculated between the inflection points (IP1: a point at which alteration in binding energy commences at the reference level; IP2: a midpoint of the Fermi energy distribution) [69]. As a result, values of 7.52, 6.83, and 7.14 eV were obtained for *N*-CQDs, TiO_2_, and *N*-CQDs/TiO_2_, respectively (Figures 7a–7c).

Upon interface formation, TiO_2_ with a lower work function compared to *N*-CQDs became conducive to electron donation. Consequently, the *N*-CQDs accepted electrons until the Fermi level reached equilibrium. An internal electric field (IEF) was generated via this charge distribution at the interfaces, leading to upward bending of the band edges of TiO_2_ and downward bending of those of the *N*-CQDs, as depicted in Figure 7d [70]. This spatial mismatch resulted in a difference in CB and VB between the *N*-CQDs and TiO_2_, facilitating the necessary charge separation [71]. During UVA irradiation, the recombination of photogenerated electrons in the CB of *N*-CQDs and holes in the VB of TiO_2_ was facilitated by the IEF and band bending. This allowed electrons in the TiO_2_’s VB and holes in the *N*-CQDs’ CB to migrate easily, fostering the interfacial connection. Consequently, a characteristic S-scheme heterojunction form aided in photogenerated charge separation through the dynamic redistribution of charge carriers around the heterogeneous interface [72]. These findings align with the superior photocatalytic activity of *N*-CQDs/TiO_2_, highlighting their exceptional charge separation characteristics.

### 3.5. Effect of various scavengers

Photogenerated holes (h^+^), hydroxyl radicals (OH^*^), and superoxide radicals $O_{2}^{-.}$ are the main ROS involved in the photocatalytic degradation of pollutants [73]. To determine the contribution of these ROS to the photocatalytic degradation of CIP in the *N-*CQDs/TiO_2_/CIP solution system, experiments were carried out with some selected scavengers under optimum conditions. The obtained results are illustrated in Figure S9. The ratio of CIP to scavenger was kept constant at 1:1 in the experiments. For this purpose, various scavenger agents including isopropanol (IPA), benzoquinone (BQ), potassium iodide (KI), sodium oxalate (Na_2_C_2_O_4_), and EDTA-Na_2_ were added to the reaction solution to act as the h^+^ and $OH_{free}^{•}$ radical scavenger, $O_{2}^{-.}$ radical scavenger, $OH_{free}^{•}$ and $OH_{surface}^{•}$ radical scavenger, and h^+^ scavenger, respectively [52, 73,74]. As seen in Figure S9, after 120 min, the percentage degradation of CIP was reduced from 83.91% to 66.04%, 49.07%, 41.40%, 31.04%, and 27.59% in the presence of IPA, BQ, KI, Na_2_C_2_O_4_, and EDTA-Na_2_, respectively. When the results obtained from the experiments were taken into account, it was understood that the ROS sequence that is effective in CIP degradation is ${\text{h}}^{+}>OH_{surface}^{•}>O_{2}^{-.}$.

**Figure 8:**
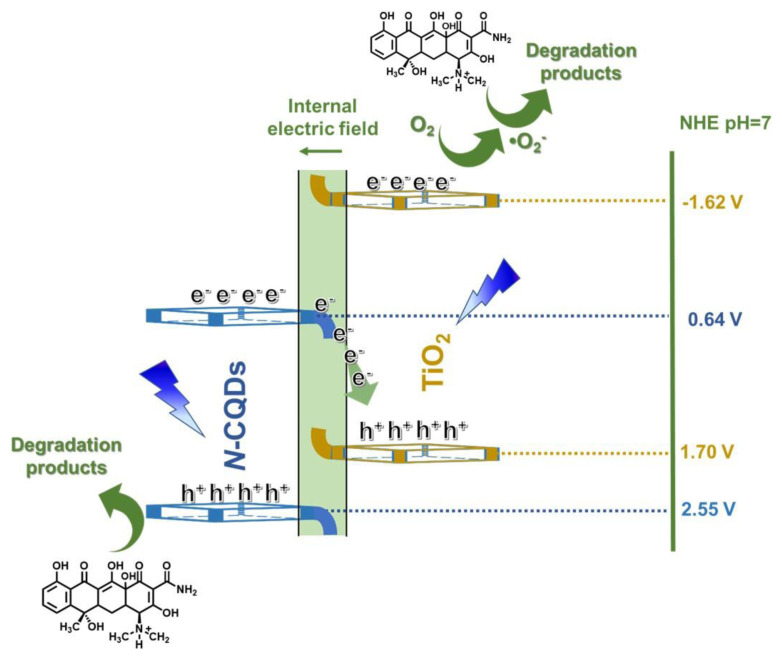
A schematic illustration of the photocatalytic mechanism for the photodegradation of CIP in the presence of *N*-CQDs/TiO_2_ nanocomposite.

Considering the results of the experiments presented so far, the proposed mechanism for the improved charge separation and increased photocatalytic activity of the *N-*CQDs/TiO_2_ photocatalyst is presented in Figure 8. The coupling that occurs by the hybridization of the conduction band of TiO_2_ with the π electrons of *N-*CQDs reduced the band gap energy of the composite formed compared to TiO_2_ and provided more radiation absorption than TiO_2_ due to the newly developed energy levels. These energy levels allowed the *N*-CQDs to act as very good electron acceptors [75]. In addition to increasing the light absorption capacity, the use of the maximum band potential of the nanocomposite with the S-scheme mechanism provides effective charge separation and enables the necessary redox reactions to occur. As seen in Figure 7d, the electrons produced by the *N*-CQDs with UVA rays recombined with the photogenerated holes of TiO_2_. Thus, while photooxidation reactions occurred in the CB of *N*-CQDs, photoreduction reactions occurred in the VB of TiO_2_.

Possible reactions during CIP degradation are given below:


(6) $$N-CQDs/{TiO}_{2}+h\nu \to e^{-}+h^{+}$$

After the formation of the photogenerated e^−^/h^+^ pair, O_2_ molecules adsorbed in the CB of TiO_2_ captured the photogenerated electrons and formed superoxide radicals ( $O_{2}^{-.}$):


(7) $$O_{2}+e^{-}\to O_{2}^{.-}$$

CIP molecules were degraded by ·O_2_^−^ radicals, but since they are unstable in aqueous solution the superoxide radicals that could not interact with CIP molecules were converted into OH* radicals according to the following reactions [76]:


(8) $$O_{2}^{.-}+2H^{+}+e^{-}\to H_{2}O_{2}$$

(9) $$H_{2}O_{2}+e^{-}\to O{H^{•}}_{surface}+{OH}^{-}$$

According to the results of the trapping experiments, since adsorbed OH* radicals are more effective in CIP degradation, the OH* radicals formed are adsorbed on the surface of the catalyst and degrade the CIP molecules. On the other hand, holes (h^+^) in the VB of the *N*-CQDs can directly destroy the CIP molecules or combine with H_2_O and turn into OH^*^ radicals:


(10) $$h^{+}+CIP\to Degradation\;products\to {CO}_{2}+H_{2}O$$

(11) $$h^{+}+H_{2}O\to {OH}^{•}+H^{+}$$

(120) $${{OH}^{•}}_{surface}+CIP\to {OH}^{•}+H^{+}$$

(13) $$O_{2}^{.-}+CIP\to Degradation\;products\to {CO}_{2}+H_{2}O$$

To compare the results of the present study with those of previous studies on CIP degradation using various catalysts, information is presented in Table S3 regarding concentration, reaction time, and CIP degradation efficiency. In comparison to the results of the studies reported in Table S3, it is understood that the presented *N*-CQDs/TiO_2_ photocatalysts showed good performance in CIP removal under the studied conditions.

## 4. Conclusion

*N*-CQDs prepared by a simple green hydrothermal technique were anchored to the surfaces of TiO_2_ nanoparticles to yield the *N-*CQDs/TiO_2_ binary heterojunction for improving the photocatalytic performance of pristine TiO_2_. Comprehensive characterization studies revealed that *N-*CQDs with an average size of 7–8 nm were successfully incorporated into the structure of the TiO_2_ nanoparticles and the *N-*CQDs/TiO_2_ binary heterojunction was successfully fabricated. As-prepared *N*-CQDs/TiO_2_ heterojunction photocatalysts exhibited good performance in the photodegradation of CIP in aqueous solutions under UVA radiation. Detailed experimentation revealed that the amount of *N*-CQDs in the *N-*CQDs/TiO_2_ nanocomposite had an important effect on photocatalytic CIP degradation, with the best combination being pH = 5 (i.e., the natural CIP pH), 0.4 g/L catalyst dose, and 10 mg/L CIP concentration whereby the ROS produced during photocatalysis played an active role in CIP degradation according to the sequence of ${\text{h}}^{+}>OH_{surface}^{•}>O_{2}^{-.}$. Under the predetermined optimum conditions, 83.91% CIP removal was achieved in 120 min. The kinetic analysis results showed that CIP removal conformed to the pseudo-first-order kinetic model. The increased photocatalytic activity of *N*-CQDs/TiO_2_ photocatalysts compared to pristine TiO_2_, thanks to the formation of the S-scheme heterojunction structure, allowed the interface formed between the *N-*CQDs and TiO_2_ to support electron transport, increased light absorption ability resulting from the quantum size effect, and reduced the tendency to resist charge transfer. As a result, this study has presented an applicable approach for the fabrication of S-scheme heterojunctions via the formation of strong interactions such as Ti-O-C. The good performance of *N*-CQDs/TiO_2_ photocatalysts in this study sheds new light on the design of efficient photocatalysts for the removal of CIP and similar organic contaminants.

## Supporting Information

### Materials

Chitosan sample with 75% deacetylation degree (DD) of ca.was bought from Sigma-Aldrich Co. (USA). Glycerol (C_3_H_8_O_3_, 99.5) from Tekkim, acetic acid (CH_3_COOH, ≥99.5%) and Urea (H_2_N-CO-NH_2_, 99%) from Merck, titanium(IV) ethoxide (TiO_2_, >99%), ethanol (C_2_H_5_OH, 99%), and ciprofloxacin (CIP, 96%) from Sigma Aldrich, hydrochloric acid (HCl, 37%) from Riedel-De-Haën were purchased. Millipore Milli-Q deionized water with properties of 20 μs/cm, approximate ionic concentration Type 3 water of 10 mg/L and 25 °C was used from Millipore Direct Q 8uv (Millipore, U.S.A.) in all experiments. The characteristics and chemical structure of Ciprofloxacin (CIP) are illustrated in Table S1.

### 2. Instrumentation

Scanning electron microscopy (SEM, Zeiss Sigma 300,Germany) and transmission electron microscopy equipped with EXA- LENS (TEM, Hitachi HT7700 TEM, Japan) with an actuated at 120 kV were used to characterize the morphologies of *N-*CQDs, TiO_2_ and *N-*CQDs/TiO_2_ samples, and the energy-dispersive X-ray spectroscopy (EDX, Zeiss, Germany) was used for elemental analysis. The powder X-ray diffraction (XRD) patterns have been recorded on a Rigaku Advanced Powder X-ray Diffraction meter operating at 30 kV and 30 mA with CuKα radiation in the 2θ of 20–80° range (0.154051 nm) to examine the crystal structure of the synthesized samples. The chemical composition and the oxidation state of the elements in the as-prepared catalyst samples was tested by X-ray Photo Electron Spectroscopy (XPS, Thermo K-Alpha). To calculate the binding energy adjustment, the C1s peak (284.5 eV) was used as a reference peak. The indium tin oxide (ITO) surface was coated with *N*-CQDs via the drop-casting method. Fourier transform infrared spectra (FT-IR) were obtained by a Tensor 27 Bruker spectrometer (Germany) employing KBr pellets with a scanning range from 4000 to 400 cm^−1^. N_2_ nitrogen adsorption-desorption isotherms at 77K were performed on a Micromeritics 3 Flex instrument (Micrometrics Instruments, USA). The surface areas of as-prepared samples were computed by the Brunauer–Emmett–Teller (BET) method, and the pore size distributions were determined by the Barrett–Joyner–Halenda (BJH) method from the desorption branch of the isotherms. The removal efficiency of CIP measurements was performed with Varian Cary 100 UV-VIS Spectrophotometer device (Varian Cary 100, Australia). Photoluminescence (PL) spectra of as-prepared samples were measured using a Shimadzu RF-5301PC spectrofluorophotometer by excitation at 325 nm with a 150 W Xe lamp. The zero-charge point (zpc) of *N*-CQDs/TiO_2_ nanocomposite was found by Malvern Zetasizer Nano ZSP (Malvern Inst.Ltd., UK). A Shimadzu UV-2550 spectrophotometer (Japan) was used to record UV–vis DRS of the synthesized samples.

### 3. Synthesis of TiO_2_

0.3 g of urea and 1.6 mL of titanium(IV) ethoxide were drop wisely added to the concentrated HCl solution mixed with 25 mL of water, and after mixing again, the mixture was transferred to the Teflon lined stainless reactor and kept at 150 °C for 6 hours. The reactor content, brought to ambient temperature, were centrifuged at 9000 rpm for 10 min. The solid part was separated and dried at 80 °C for 7–8 h. This solid product was calcined in a muffle furnace at 300°C for 2 hours.

### Experimental device and the procedure

The experimental device for photocatalytic degradation of ciprofloxacin was conducted in a magnetically stirred quartz cylindrical reactor with a working volume of 500 mL (reactor vessel dimensions 50.0 mm × 250.0 mm, Çalışkan Cam, Turkey). Magnetic stirring was used to achieve effective interaction between the catalysts and the organic contaminate. The outer surface of the reactor was completely covered with aluminum foil to obtain maximum efficiency from the UV source. 16 W UV-A (Sylvania, Japan) was used as the UV irradiation source. Batch studies were carried out with the constant CIP solution of 500 mL to determine the effects of various processing variables, catalyst dosage (0.05–0.60 g/L), initial CIP concentration (5–25 mg/L) and pH (2–10), on the degradation efficiency of CIP. The pH value was adjusted by adding 0.1 M HCl or NaOH solution using a pH meter (Mettler Toledo, China). The suspension was then agitated in dark for 20 min to reach the equilibrium. Afterward, the UV-A lamp was placed into the reactor and turned on. In addition, adsorption experiments conducted without UV irradiation were also performed in covered beakers to ensure similar processing conditions with the photo-catalytic experiments. At the predetermined time intervals, approximately 3 mL solution was taken and then centrifuged at 5000 rpm for 4 min. In order to stop the photocatalytic reactions in the filtrate, 0.5 mL methanol was added to the solution. The remaining CIP concentration was finally measured using a Varian Cary 100 UV–vis spectrophotometer at the maximum wavelength of 276 nm. The degradation efficiency (%) of CIP was calculated from the below equation:

$$Degradation\;efficiency=\left[\frac{A_{0}-A_{t}}{A_{0}}\right]\times 100$$

in which A_0_ and A_t_ exemplify the CIP absorbance values for the initial and after t period (min).

#### Supplementary Data

Figure S1 Synthesis flowchart of *N-*CQDs/TiO_2_ nanocomposites.

#### 5.Catalyst characterization

Figure S2 SEM images of *N*-CQDs with different magnification (A, B), bare TiO_2_ (C) *N-*CQDs/TiO_2_ nanocomposite (D), and their EDX spectrum (E)

Figure S3 (a) XPS survey spectra of as-prepared *N*-CQDs, TiO_2_, and *N*-CQDs/TiO_2_ nanocomposite and (b) high resolution XPS N 1s spectra for *N*-CQDs

Figure S4 **(a)** N_2_ adsorption*-*desorption isotherms for *N-*CQDs, TiO_2_, and *N-*CQDs/TiO_2_ nanocomposites, **(b)** BJH pore size distribution of the corresponding materials.

#### 6.Photocatalytic CIP Degradation

Figure S5 Photocatalytic degradation of CIP at different *N-*CQDs/TiO_2_ loading. Experimental conditions: [CIP]_0_= 10 mg /L, and pH=5.

Figure S6 The variation of CIP degradation with initial CIP concentration and reaction time. Experimental conditions: [*N*-CQDs/TiO_2_]_0_= 0.4 g/L, and pH=5.

Figure S7 **(a)** Impact of initial solution pH. Experimental conditions : [CIP]_0_ = 10 mg/L, and [Catalyst]_0_ = 0.4 g/L **(b)** Zero point of charge (pH_zpc_) for *N*-CQDs/TiO_2_

Figure S8 VB-XPS analyses of (a) *N*-CQDs (b) TiO_2_ (c) band alignments of *N*-CQDs, and TiO_2_.

Figure S9 Impact of scavengers. Conditions: [CIP]_0_ = 10 mg/L, and [Catalyst]_0_ = 0.4 g/L, [Scavenger]_0_ = 10 mg/L, and pH = 5.

**Table S1:** Structure and characterization of Ciprofloxacin (CIP)

Chemical structure	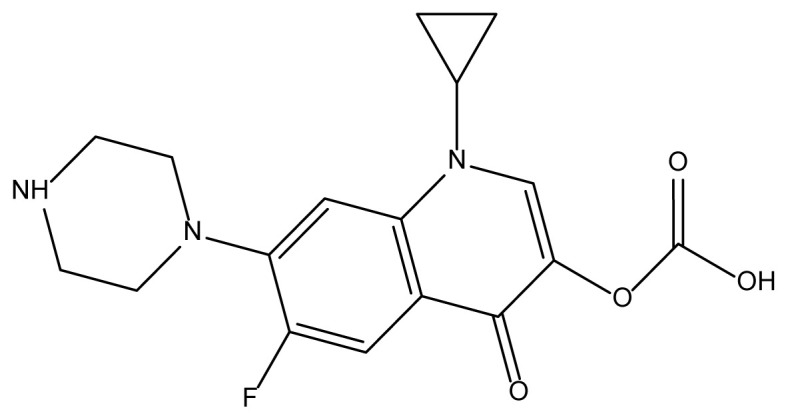
Molecular formula	C_17_H_18_FN_3_O_3_
Mw g/mol)	331.346
λmax (nm)	276
Solubilitiy in water (mg/mL)	30
Therapeutic group	Antibiotic

**Table S2:** Textural characteristics of the as-synthesized materials of TiO_2_, *N-*CQDs, *N-*CQDs/TiO_2_

Parameter	TiO _2_	*N-*CQDs	*N-*CQDs/TiO _2_
BET surface area (m ^2^/g)	71.798	1.091	213.792
BJH cumulative surface area (m ^2^/g)	78.025	2.367	252.690
Total pore volume (cm ^3^/g) ^(a)^	0.186	0.003	0.203
BJH Desorption average pore width (nm) ^b^	9.524	5.171	3.210

a Obtained by the BJH method.

b Computed by the BJH (desorption) method using N_2_ adsorption isotherm.

**Table S3:** Comparison of the CIP degradation efficiencies with reported different photocatalysts

Photocatalysts	Catalyst loading (g/L)	CIP concentration (mg/L)	Reaction time (min)	Degradation efficiency (%)	References
MIL100(Fe)@DPANI@CelF	0.25	32	180	82.78	[ 1]
ZnO	0.02	5	60	48	[ 2]
NiS/MoS _2_/C _3_N _4_	1.00	10	120	71.3	[ 3]
BiOCl	0.25	10	240	74	[ 4]
Sepiolite/g-C _3_N _4_/Pd	0.40	10	60	64	[ 5]
Co-BiOCl/CQDs	0.50	20	100	79.6	[ 6]
CQDs/PbBiO _2_Cl	0.30	10	75	78.9	[ 7]
*N-*CQDs/TiO _2_ nanocomposite	0.40	10	120	83.91	This work

References1

HouX
SunL
HuY
AnX
QianX

De-doped polyaniline as a mediating layer promoting in-situ growth of metal–organic frameworks on cellulose fiber and enhancing adsorptive-photocatalytic removal of ciprofloxacin
Polymers
2021
13
19
3298
10.3390/polym13193298
34641114
PMC85121022

El-KemaryM
El-ShamyH
El-MehassebI

Photocatalytic degradation of ciprofloxacin drug in water using ZnO nanoparticles
Journal of Luminescence
2010
130
12
2327
2331
10.1016/j.jlumin.2010.07.013
3

LuX
WangY
ZhangX
XuG
WangD


NiS and MoS_2_ nanosheet co-modified graphitic C_3_N_4_ ternary heterostructure for high efficient visible light photodegradation of antibiotic
Journal of Hazardous Materials
2018
341
10
19
10.1016/j.jhazmat.2017.07.004
28763632
4

SenasuT
NarenuchT
WannakamK
ChankhanitthaT
NananS

Solvothermally grown BiOCl catalyst for photodegradation of cationic dye and fluoroquinolone-based antibiotics
Journal of Materials Science: Materials in Electronics
2020
31
9685
9694
10.1007/s10854-020-03514-4
5

ChuaichamC
PawarRR
KarthikeyanS
OhtaniB
SasakiK

Fabrication and characterization of ternary sepiolite/g-C_3_N_4_/Pd composites for improvement of photocatalytic degradation of ciprofloxacin under visible light irradiation
Journal of Colloid and Interface Science
2020
577
397
405
10.1016/j.jcis.2020.05.064
32502666
6

LiW
HuangJ
FuX
XuJ
YuX


CQDs modified Co-BiOCl nanosheets with improved effective light absorption and charge separation for photocatalytic CIP degradation and NOX removal
Surfaces and Interfaces
2021
27
101541
10.1016/j.surfin.2021.101541
7

ShengY
YiD
QingsongH
TingW
MingL


CQDs modified PbBiO_2_Cl nanosheets with improved molecular oxygen activation ability for photodegradation of organic contaminants
Journal of Photochemistry and Photobiology A: Chemistry
2019
382
111921
10.1016/j.jphotochem.2019.111921
