# Supplementary figures and images for: Exploring the efficiency of nitrogenated carbon quantum dots/TiO2 S-scheme heterojunction in the photodegredation of ciprofloxacin in aqueous environments
Source: Turk J Chem. 2024 Mar 11;48(4):550–67. doi: 10.55730/1300-0527.3679 (PMC11407339; doi:10.55730/1300-0527.3679)

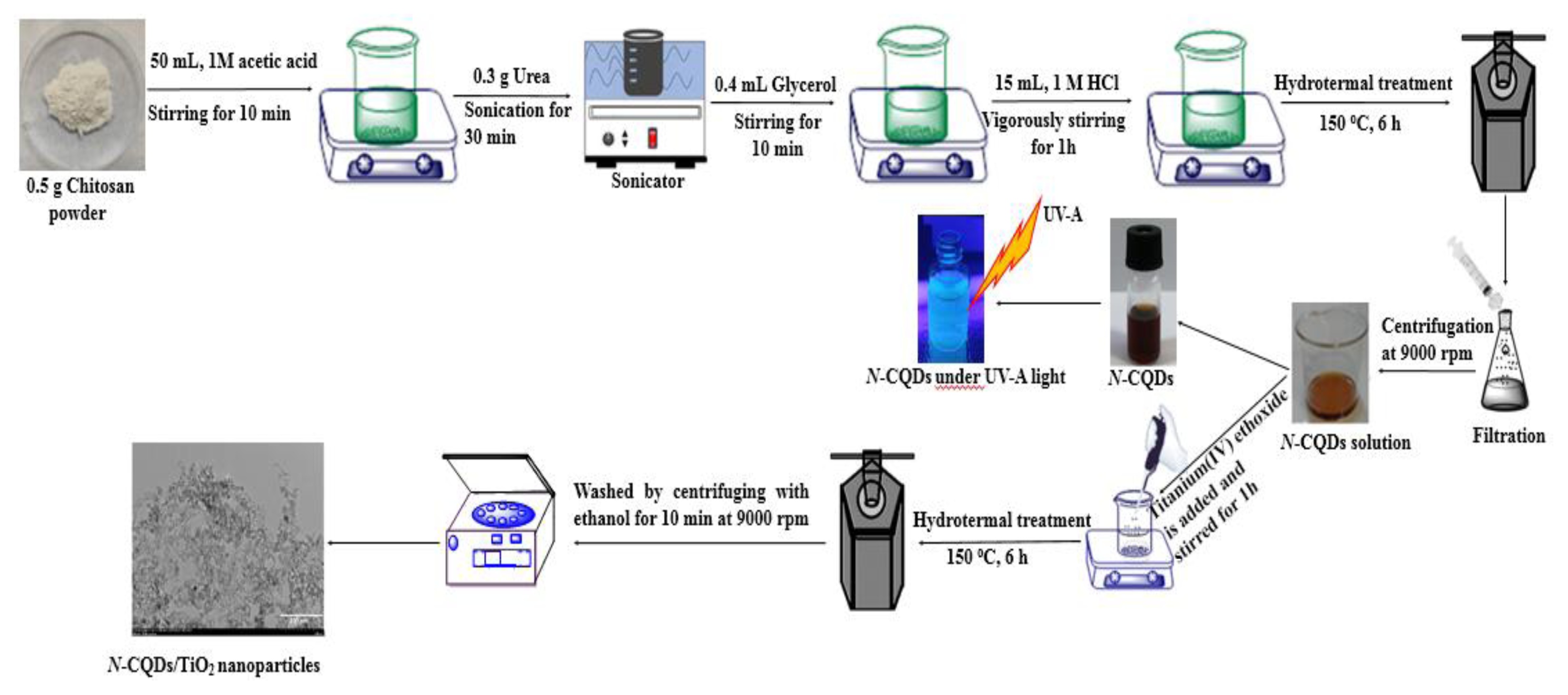

Supplement: Figure S1 — Synthesis flowchart of N-CQDs/TiO2 nanocomposites. [file tjc-48-04-550s1.tif]

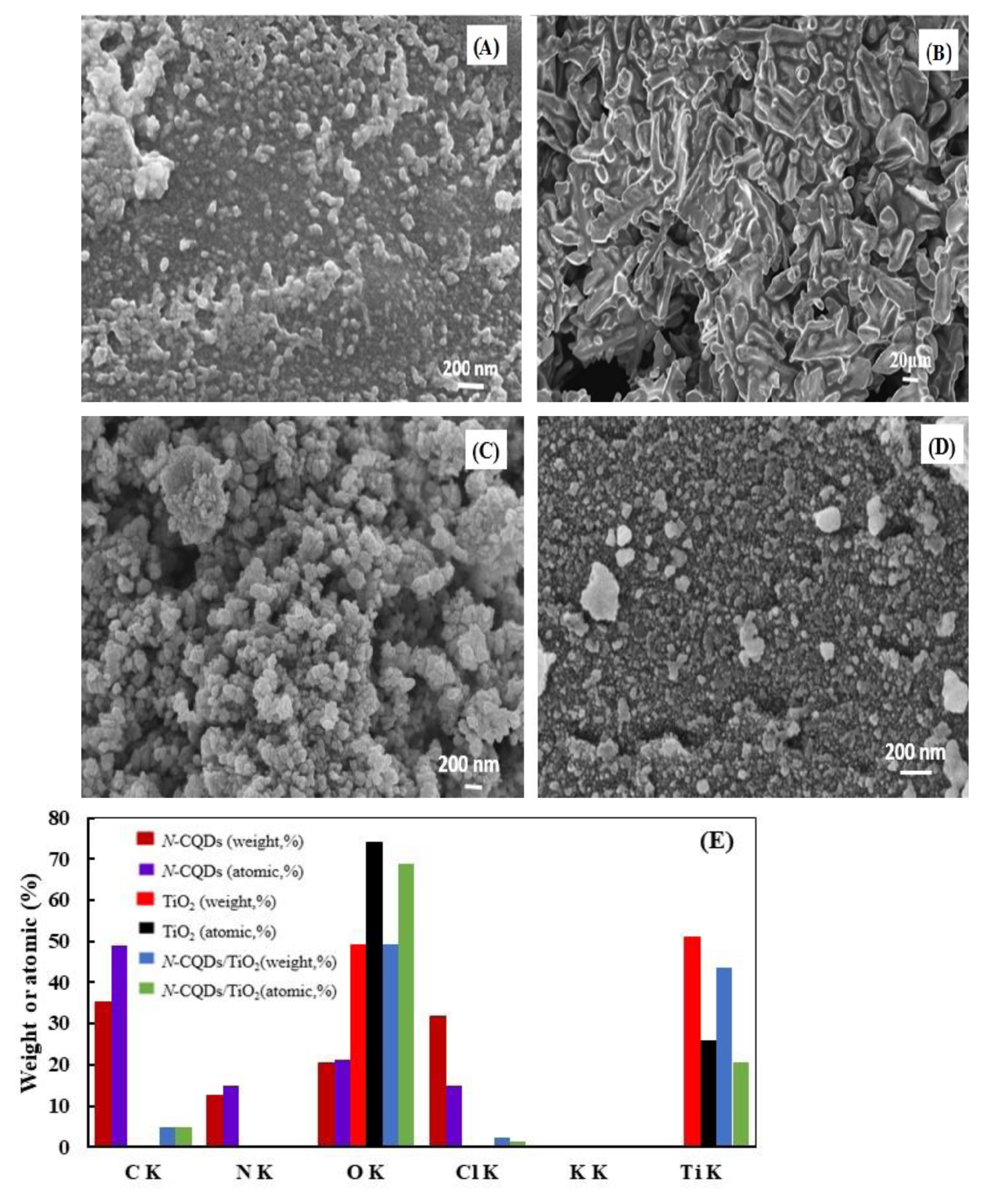

Supplement: Figure S2 — SEM images of N-CQDs with different magnification (A, B), bare TiO2 (C) N-CQDs/TiO2 nanocomposite (D), and their EDX spectrum (E) [file tjc-48-04-550s2.tif]

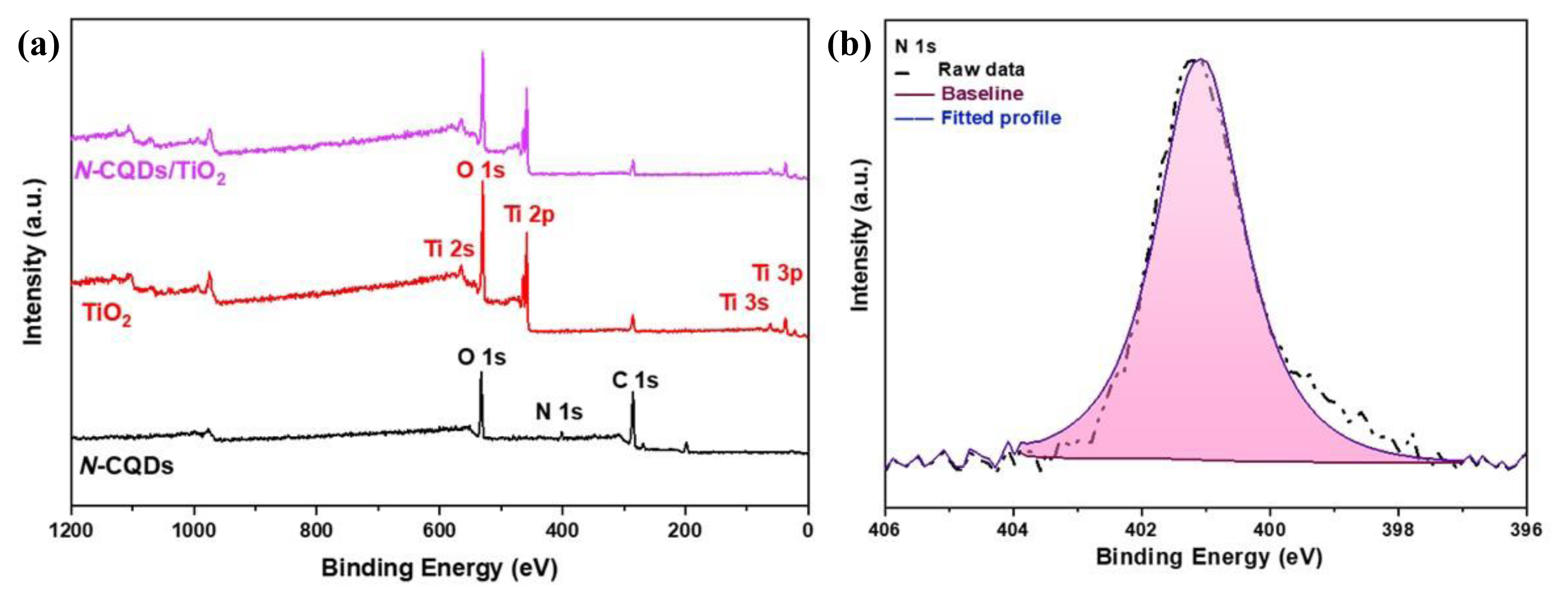

Supplement: Figure S3 — (a) XPS survey spectra of as-prepared N-CQDs, TiO2, and N-CQDs/TiO2 nanocomposite and (b) high resolution XPS N 1s spectra for N-CQDs [file tjc-48-04-550s3.tif]

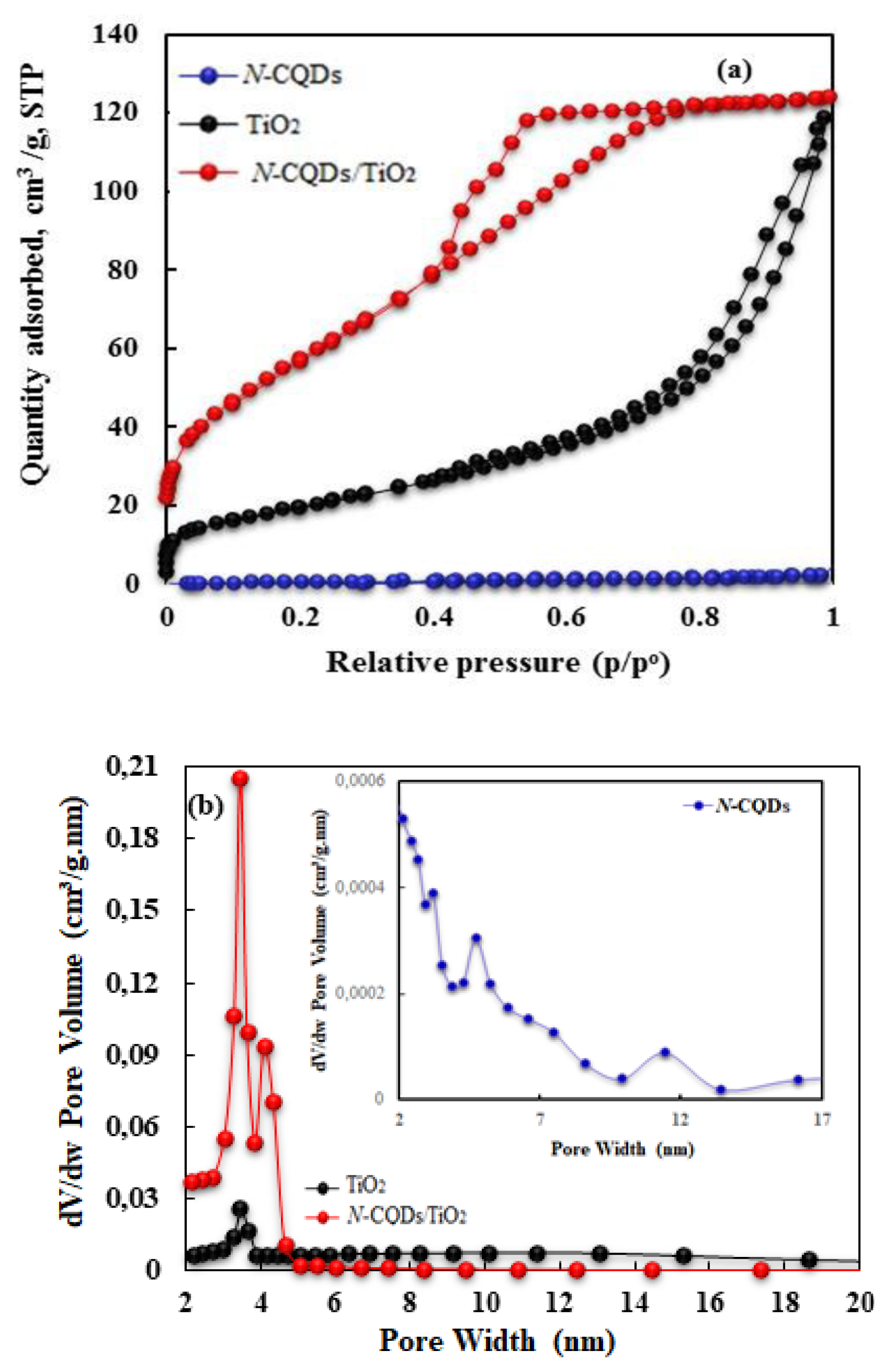

Supplement: Figure S4 — (a) N2 adsorption-desorption isotherms for N-CQDs, TiO2, and N-CQDs/TiO2 nanocomposites, (b) BJH pore size distribution of the corresponding materials. [file tjc-48-04-550s4.tif]

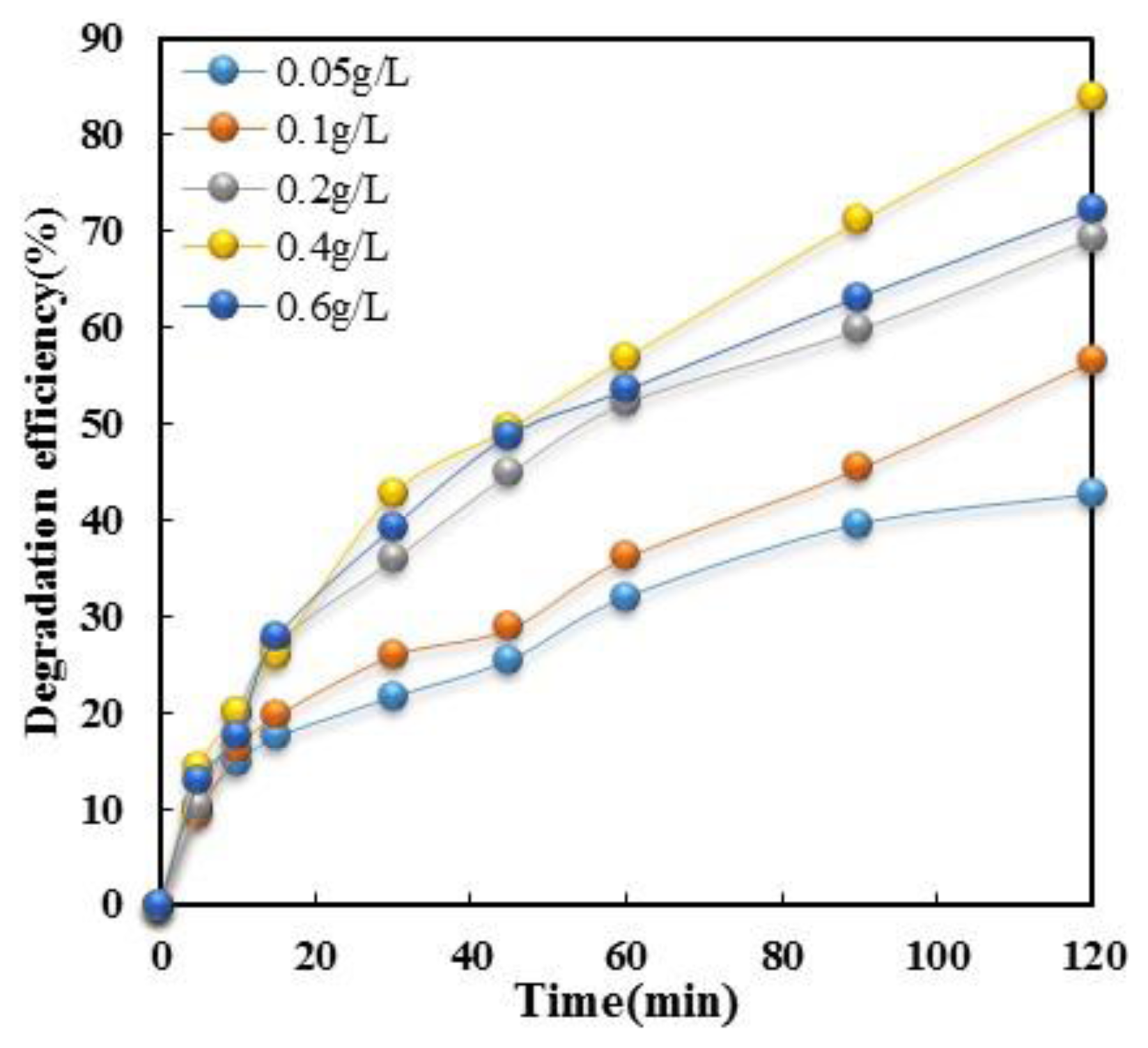

Supplement: Figure S5 — Photocatalytic degradation of CIP at different N-CQDs/TiO2 loading. Experimental conditions: [CIP]0= 10 mg /L, and pH=5. [file tjc-48-04-550s5.tif]

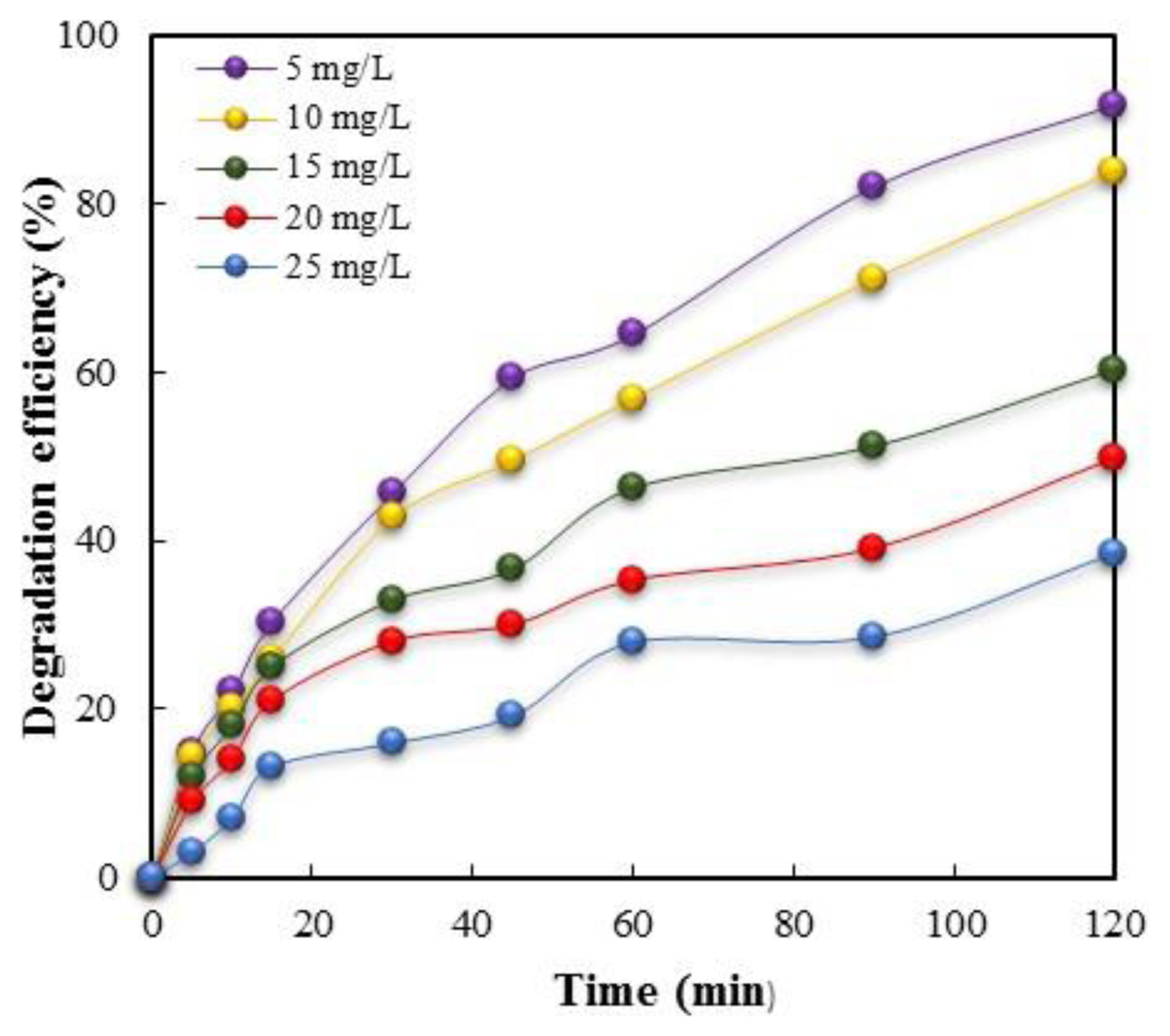

Supplement: Figure S6 — The variation of CIP degradation with initial CIP concentration and reaction time. Experimental conditions: [N-CQDs/TiO2]0= 0.4 g/L, and pH=5. [file tjc-48-04-550s6.tif]

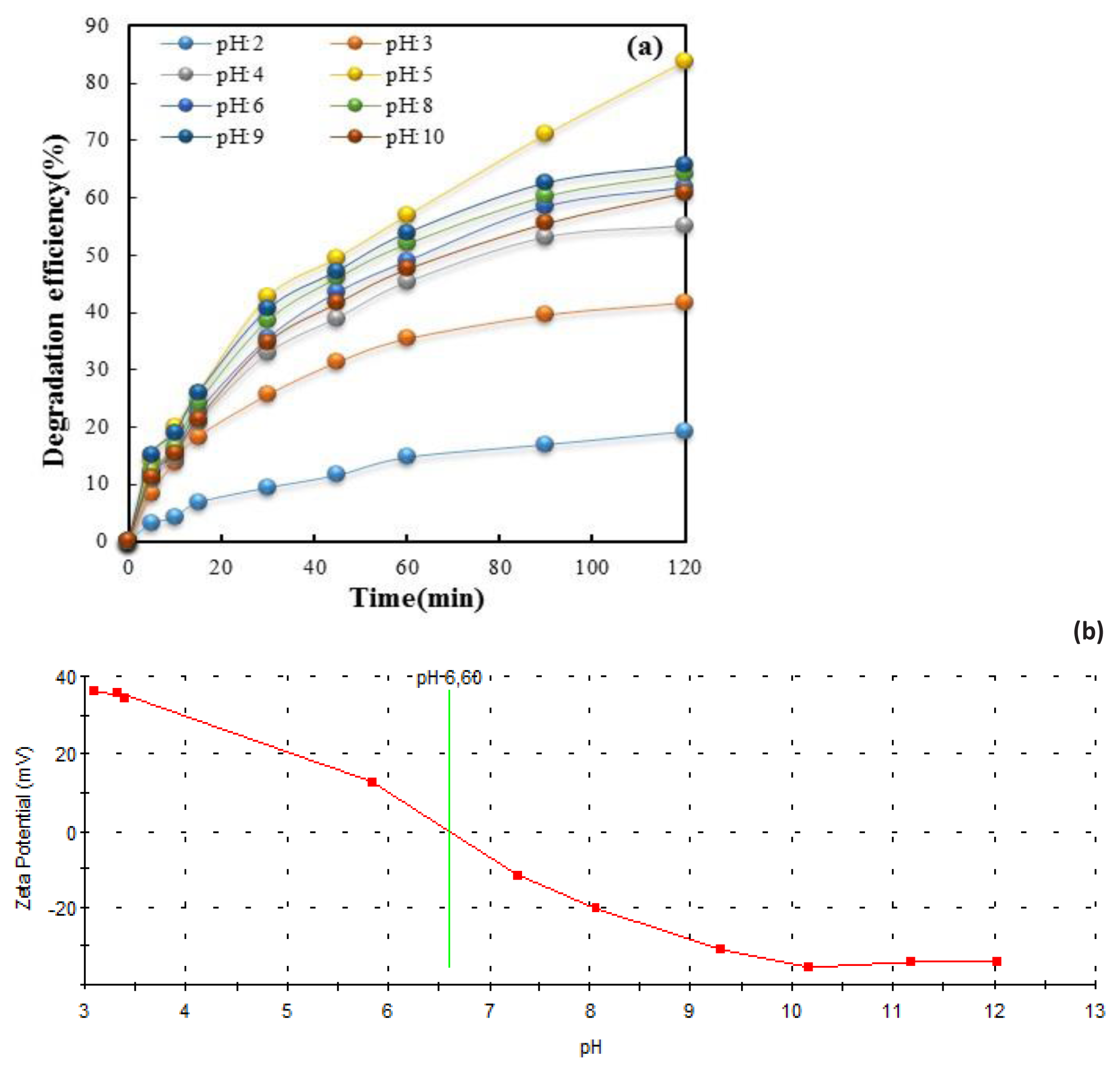

Supplement: Figure S7 — (a) Impact of initial solution pH. Experimental conditions : [CIP]0 = 10 mg/L, and [Catalyst]0 = 0.4 g/L (b) Zero point of charge (pHzpc) for N-CQDs/TiO2 [file tjc-48-04-550s7.tif]

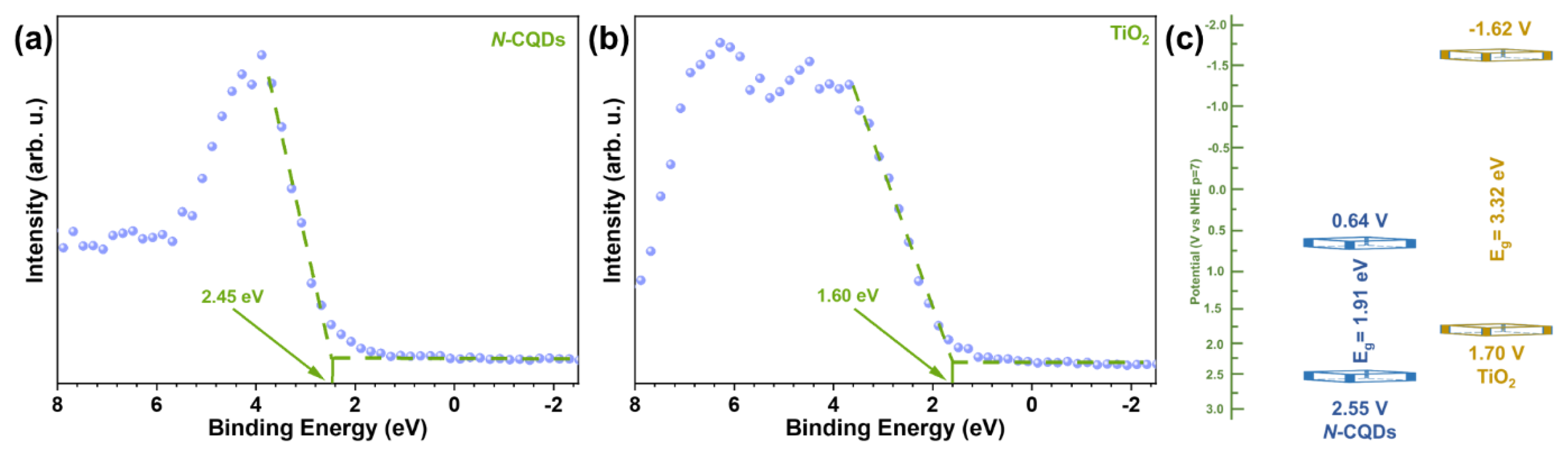

Supplement: Figure S8 — VB-XPS analyses of (a) N-CQDs (b) TiO2 (c) band alignments of N-CQDs, and TiO2. [file tjc-48-04-550s8.tif]

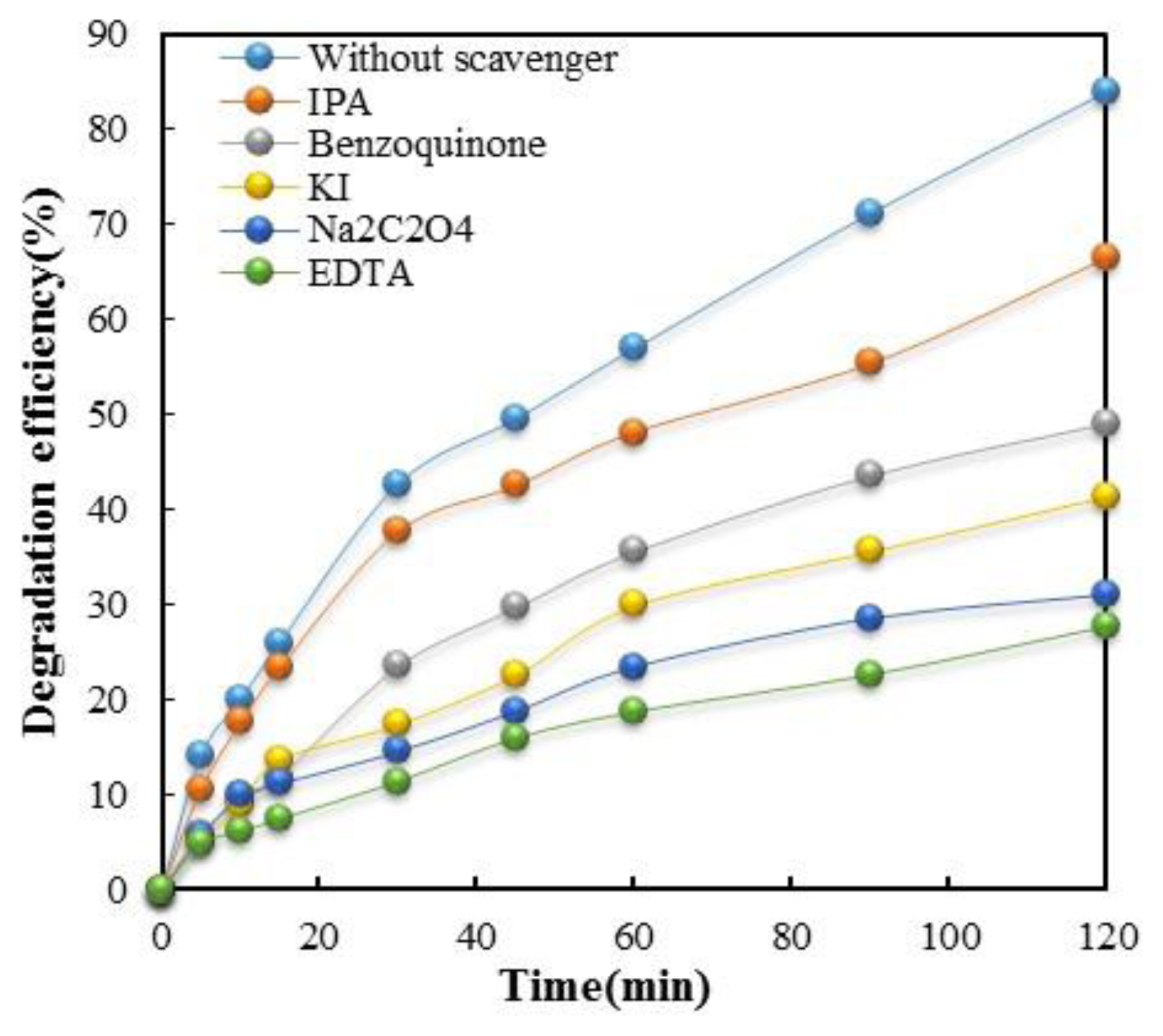

Supplement: Figure S9 — Impact of scavengers. Conditions: [CIP]0 = 10 mg/L, and [Catalyst]0 = 0.4 g/L, [Scavenger]0 = 10 mg/L, and pH = 5. [file tjc-48-04-550s9.tif]
